# Single-cell landscape dissecting the transcription and heterogeneity of innate lymphoid cells in ischemic heart

**DOI:** 10.3389/fimmu.2023.1129007

**Published:** 2023-05-09

**Authors:** Shijiu Jiang, Yuqi Zheng, Bingjie Lv, Shaolin He, Wenling Yang, Boyuan Wang, Jin Zhou, Shangwei Liu, Dazhu Li, Jibin Lin

**Affiliations:** ^1^ Department of Cardiology, Union Hospital, Tongji Medical College, Huazhong University of Science and Technology, Wuhan, China; ^2^ Hubei Key Laboratory of Biological Targeted Therapy, Union Hospital, Tongji Medical College, Huazhong University of Science and Technology, Wuhan, China; ^3^ Hubei Provincial Engineering Research Center of Immunological Diagnosis and Therapy for Cardiovascular Diseases, Union Hospital, Tongji Medical College, Huazhong University of Science and Technology, Wuhan, China; ^4^ Department of Cardiology, The First Affiliated Hospital, Shihezi University, Shihezi, Xinjiang, China

**Keywords:** innate lymphoid cells, myocardial infarction, myocardial ischemia-reperfusion injury (MIRI), single-cell RNA sequencing, transcription

## Abstract

**Background:**

Until now, few articles have revealed the potential roles of innate lymphoid cells (ILCs) in cardiovascular diseases. However, the infiltration of ILC subsets in ischemic myocardium, the roles of ILC subsets in myocardial infarction (MI) and myocardial ischemia-reperfusion injury (MIRI) and the related cellular and molecular mechanisms have not been described with a sufficient level of detail.

**Method:**

In the current study, 8-week-old male C57BL/6J mice were divided into three groups: MI, MIRI and sham group. Single-cell sequencing technology was used to perform dimensionality reduction clustering of ILC to analyze the ILC subset landscape at a single-cell resolution, and finally flow cytometry was used to confirm the existence of the new ILC subsets in different disease groups.

**Results:**

Five ILC subsets were found, including ILC1, ILC2a, ILC2b, ILCdc and ILCt. It is worth noting that ILCdc, ILC2b and ILCt were identified as new ILC subclusters in the heart. The cellular landscapes of ILCs were revealed and signal pathways were predicted. Furthermore, pseudotime trajectory analysis exhibited different ILC statuses and traced related gene expression in normal and ischemic conditions. In addition, we established a ligand–receptor–transcription factor–target gene regulatory network to disclose cell communications among ILC clusters. Moreover, we further revealed the transcriptional features of the ILCdc and ILC2a subsets. Finally, the existence of ILCdc was confirmed by flow cytometry.

**Conclusion:**

Collectively, by characterizing the spectrums of ILC subclusters, our results provide a new blueprint for understanding ILC subclusters’ roles in myocardial ischemia diseases and further potential treatment targets.

## Introduction

MI usually results from coronary artery obstruction and can cause insufficient myocardium blood supply. Though substantial progress in the therapy of MI, particularly in the early reperfusion of the criminal coronary artery using percutaneous coronary intervention (PCI), MIRI can cause serious damage to the original ischemic myocardium and remains a significant challenge in clinical settings without clear mechanisms. A great deal of research has focused on the mechanisms of myocardial ischemia and reperfusion- associated tissue injury in recent decades. Additionally, a growing body of evidence suggests that inflammation and immune response play important roles in these conditions (1–3).

ILCs are a newly discovered group of lymphocytes that lack the rearranged antigen receptors expressed by T and B cells and were recently found to be involved in innate immunity (4). ILCs can be divided into three groups according to their transcription factor profiles and their cytokine secretion: Group 1, Group 2 and Group 3 ILCs. Group 1 ILCs, including ILC1s and natural killer (NK) cells, produce IFN-γ and express the T box transcription factor (T-bet). ILC1s can react to intracellular pathogens and promote type 1 immune response (5). Group 2 ILCs, whose development and function depend on retinoic acid receptor-related orphan receptor-α (RORα) and GATA binding protein 3 (GATA3), can produce type 2 cytokines, predominantly IL-13 and IL-5. ILC2s can act on allergens and extracellular parasites and participate in the repair of tissue and organ damage (6). Group 3 ILCs, including ILC3s and lymphoid tissue- inducer (LTi) cells, can produce IL-22 and IL-17 and are dependent on RORγt. ILC3s can defend against bacteria, fungi and other extracellular microorganisms, and are involved in maintaining intestinal stability (7). ILCreg, a potential new subset of ILCs expressing specific transcription factor Id3 and secreting IL-10, was identified in the intestine and can control innate intestinal inflammation (8).

Recently, several ILC members, mainly ILC2s, were shown to be implicated in cardiovascular diseases such as atherosclerosis, pericarditis, cardiac fibrosis and MI (9–13). The distribution of ILC2s was found in the heart and the surrounding tissues, including the aorta, perivascular adipose tissue and lymph nodes. It was reported that the proportion of ILC1s was increased while ILC2s were decreased in a human acute ST-segment elevation myocardial infarction (STEMI) patient (14). Interestingly, Yu et al. demonstrated that ILC2s expression was increased after MI (13). Furthermore, they found that ILC2s could promote cardiac healing and ventricular function recovery after MI. Using low- dose IL-2 to activate ILC2 may be a new therapeutic strategy to promote the repair response after myocardial infarction. Chen et al. found that IL-33 treatment expanded cardiac ILC2s and showed a protective effect on cardiac tissue damage, reducing myocardial cell death, immune cell infiltration, tissue fibrosis and improving cardiac function (12). It is worth noting that there is currently no research directly studying ILCs in MIRI to the authors’ knowledge. However, there are some reports characterizing ILCs in ischemia-reperfusion injury to other organs, such as ILC1s and ILC3s in liver ischemia-reperfusion injury (IRI) (15, 16) and ILC2s and ILCreg in renal IRI (17, 18). Furthermore, ILC- related cytokines were found to play important roles in MIRI and MI (19).

However, due to the unique heterogeneity and plasticity of ILCs, the roles and regulation modes of various ILC subsets in MI, and in MIRI especially, are still unclear. Furthermore, the development and differentiation of ILC subsets are still not fully understood. sc-RNA- seq technology allows the analysis of gene expression at single-cell resolution, can identify complex and rare cell subsets, reveal the regulatory networks among genes, uncover communications among cells and track cell development trajectories (20). For example, scRNA-seq was used in psoriasis and revealed skin-resident ILCs converging on a pathogenic effector state (21). Additionally, scRNA-seq identified a PD-1^hi^ ILC progenitor from mouse bone marrow progenitors and defined its development pathway (22). One very recent study using multiple omics techniques provided an integrative molecular map of human myocardial infarction (23). Importantly, there are currently very few sets of sequencing data for ILCs in the heart. Hence, scRNA-seq for ILCs in MI and MIRI may aid further understanding of the roles of ILC subsets in ischemic myocardium.

In this mouse study, the cellular landscapes of ILCs in sham, MI and MIRI groups were revealed. Five ILC subsets were identified, including ILC1, ILC2a, ILC2b, ILCdc and ILCt. We specifically highlight the analysis for ILCdc here. Pseudotime trajectory analysis exhibited gene expression in different ILC statuses. In addition, cell communications among ILC clusters were disclosed. Moreover, we further dissected the landscape of subsets of ILCdc and ILC2a with single-cell reclustering analysis. Finally, we confirmed the existence of ILCdc using flow cytometry. The ultimate purpose of our study was to depict the landscape of ILC clusters in myocardial ischemia diseases and provide potential treatment targets.

## Materials and methods

### Animals

Male C57BL/6J mice aged 8 weeks were purchased from Shulaibao Biotechnology Co., Ltd. (Wuhan, China) and housed in a specific pathogen-free facility in Animal Center of Tongji Medical College, Huazhong University of Science and Technology. All animal studies were performed according to the principles of the National Institutes of Health Guide for the Care and Use of Laboratory Animals and approved by the Animal Care and Use Committee of Union Hospital, Huazhong University of Science and Technology, China.

### Mice MI/MIRI/sham model

Mice ischemia models were performed according to the protocol we previously described (24). Briefly, after being anesthetized by intraperitoneal injection (1% sodium pentobarbital solution, 50mg/kg), the mice were intubated and connected to a ventilator. Then, the heart was exposed to find the left anterior descending coronary artery (LAD) under a stereo microscope, and the LAD was then ligated at about 1-2mm below the left atrial appendage with a 6-0 suture needle. For the MI model, the anterior wall of the left ventricle below the ligation line turned pale, and the ligation line was not released, while for the MIRI model, the ligation wire was released after 30 minutes, and the ventricular wall below the ligation point gradually returned to pink. The silk thread was passed under the LAD but without ligation for sham models. Finally, the thorax was closed and the mice fed for 3 days. We gave 100ul of buprenorphine (0.1mg/mL, subcutaneous injection) to relieve the pain of the mice when they returned to autonomic respiration after the operation, and additional doses were given every 4-6 hours for 24 days after surgery. In addition, ibuprofen (0.2 mg/mL) was added to drinking water 2 days before surgery and ≤3 days after surgery.

### Preparation of cardiac mononuclear cell suspension

Three days after operation, the mice were sacrificed by cervical dislocation, and the hearts were quickly removed after the blood in the ventricular chambers was washed using PBS. Then, the infarct area in the left ventricle of the heart was chopped up and digested with collagenase B (1mg/ml) according to the product instruction. RPMI 1640 medium supplemented with 10% FBS was used to stop the enzyme digestion reaction. Then, the cardiac mononuclear cell suspension was obtained by density gradient centrifugation with Percoll solution.

### Flow cytometry and ILC sorting

The cardiac mononuclear cell suspension was separated into EP tubes with 5 × 10^6^ cells per tube; one sample from each group was selected to sort ILCs and each sample consisted of pools of ten mice. Then, FITC-labeled Mouse Lineage Antibody Cocktail, APC/Cy7 labeled anti-mouse CD45 antibody and PE-labeled anti-mouse CD127 antibody were added and incubated with the cells for 30 minutes at 4°C in the dark. ILCs were sorted on a flow cytometer (LE-SH800) and 7-AAD was added to label the dead cells.

### Single-cell RNA sequencing

The ILC cells obtained by flow sorting were subjected to single-cell RNA sequencing on the 10X Genomics DNBSEQ platform. The prepared ILC suspension was then partitioned into Gel Beads in Emulsions (GEMs) in the automated Chromium Controller, and then mRNAs were reverse- transcribed into cDNAs. A reaction system was configured for breaking GEMs; after reacting at the suitable temperature for a fixed period of time, the products were purified. The PCR reaction system was prepared and the reaction time was suitable for a certain temperature. After that, the cDNAs were subjected to fragmentation, end repair, “A” base addition and adaptor ligation. The PCR reaction system was configured. After reacting at the suitable temperature for a fixed period of time, amplification was processed *via* PCR. The circularization program was then performed to produce single-stranded cyclized products and digested DNA molecules that were uncyclized and linear. After that, rolling cycle amplification was used to replicate the single-stranded circle DNA molecules and generated DNA nanoballs (DNBs) which contained multiple copies of DNA. The high-intensity DNA nanochip technique was then used to load enough quality DNBs and combinatorial Probe-Anchor Synthesis was used for sequencing. The data generated by sequencing were collected in a FASTQ format and then aligned with the Mouse Genome (mm10) using the CellRanger pipeline (v5.0.1) (25). The data were then analyzed using Rstudio.

### Data analysis of single-cell RNA sequencing

#### Data quality control and cell screening

Low-quality cells with less than 200 or more than 6500 detected genes, and those with mitochondria gene percent exceeding 20%, were filtered out. In order to avoid cell contamination and ensure the purity of ILCs, we screened and extracted cells with positive expression of Cd127 (Il7r) and Cd45 (Ptprc). Then, the cells expressing genes Cd3e, Cd3d, Cd4, Cd8a, Cd19, Cd11c (Itgax), Cd11b (Itgam), Cma1, Ly6c1, Adgre1 (F4/80) or Pdgfra were removed to avoid contamination of T cells, B cells, dendritic cells, mast cells, monocytes, macrophages or fibroblasts.

#### Elimination of potential double cells

The R package “DoubletFinder” (26) was subjected to eliminate potential double cells. The principle of “DoubletFinder” is to artificially simulate some double cells, mix the simulated double cells with the cells in the original matrix and then perform dimensionality reduction clustering. In principle, the artificial doublets will be close to the real doublets; therefore, the proportion of artificial simulated doublets in the nearest- neighbor cells in each cell (pANN) was calculated, the probability of doublets in each barcode was ranked according to the pANN value and the number of doublets in each sample could be calculated according to the statistical principle of Poisson distribution. Combined with the previous cell pANN value, doublets can be filtered. We set the best pK value, the optimized expected doubletRate and pN to 0.29, 0.023 and 0.25, respectively, and 45 potential double cells were eliminated.

#### Normalization and PCA dimensionality reduction

R package Seurat (v 3.2.0) (27) was used for downstream analysis. After quality control, the Normalize Data function was used to normalize the data, the Integrate Data function was used to integrate each sample data point and eliminate the batch effect and the Scale Data function was used to normalize the combined data. Then, 2000 hypervariable genes were identified by the Find Variable Features function.

The principal component analysis (PCA) was further carried out, and the ElbowPlot function was used to draw the distribution point map of all PCs. Then, unbiased clustering of cells was performed by the shared nearest- neighbor (SNN) algorithm and Find Clusters function; here, we used the default parameter of SNN. Finally, Uniform Manifold Approximation and Projection (UMAP) was used to achieve visual dimension reduction.

#### Cell cycle score

To eliminate the effect of cell- cycle- related genes on dimensionality reduction clustering, we calculated the cell cycle score of each cell based on the expression of classic marker genes in the G2/M and S phases. We used the Cell Cycle Scoring function to calculate the cell cycle score of each cell, and calculated the S and G2/M phase scores to predict whether the cell was in the G2M, S or G1 phase. If the data are greatly affected by cell cycle genes, it is necessary to eliminate the effect of the cell cycle on subsequent analysis. The function “vars.to.regress” can be set to regress the cell cycle score to eliminate the effect of cell- cycle heterogeneity.

#### Differentially expressed genes identification and cell- cluster annotation

The FindAllMarkers function was used to identify genes in different ILC clusters. Those with a logFC > 0.25 and expressed in at least 25% of the cells were selected as significant marker genes. Heatmap and dotplot were used for visualization. Finally, R package “scType” (28) and ILC-related marker genes reported in the literature were used to annotate each cluster.

#### Pseudotime trajectory analysis

The R package “monocle2” with default settings was used for pseudotime trajectory analysis (29). Additionally, the differentially expressed genes identified by functional–differential GeneTest were used for pseudotime ordering. The DDRTree method was employed to achieve dimension reduction and align cells along the pseudotime trajectory. Branch analysis was performed with the BEAM function.

#### GSVA/GO/KEGG/GSEA analysis

To explore the potential biological function of ILC clusters in mice ischemic myocardium, we used the “GSVA” function (30) for pathway enrichment analysis. The “clusterProfiler” (31) function was used for GO annotation and KEGG pathway enrichment analysis. Additionally, the GSEA (32) was used to display the set of genes downstream of transcription factor regulation.

#### Inferring cell communication in ILC clusters

The communication among ILC clusters were inferred by the function CellCall (33), which integrated intercellular and intracellular signals by collecting ligand–receptor–transcription factor (L-R-TF) axis data based on the KEGG. Additionally, the target genes of transcription factors were also included.

#### Analysis of TF regulatory network

SCENIC (34) with default parameters was subjected to TF regulatory network analysis. Murine mm9 TFs were downloaded as a reference, and candidate target genes (regulons) were predicted by enriched TF-binding motifs; then, the regulon activities were inferred by RcisTarget. The AUC enrichment results of individuals were added to the Seurat object, and finally the TF–regulon interaction network was obtained and graphed by the “igraph” package of Rstudio.

#### hdWGCNA (weighted gene co-expression network analysis in high dimensional data) analysis of ILCs clusters

Since hdWGCNA analysis (35) is extremely sensitive to data sparsity, the k-Nearest- Neighbors (KNN) algorithm was employed to generate a metacell gene expression matrix to reduce data sparsity. Then, ILC subsets were extracted for co-expression network analysis. The subsets were grouped according to cluster, and the soft power threshold was set as 6. “Pearson” correlation was used to construct a topological overlap matrix (TOM), which reflects the similarity of gene expression, and the “PlotDendrogram” function was used to draw the cluster tree. After clustering into modules, ModuleEigengenes ME was used to perform correlation analysis between modules and cell types, and the most relevant modules were obtained. The highly connected genes in each module were then determined by calculating the eigengene-based connectivity (kME), whose essence was the correlation between genes and the module eigengenes. Individual modules were sorted by kME value. We selected the top 10 hub genes based on the kME value and selected the top 3 enrichment pathways based on the enrichment scores of ModuleEigengenes; finally, the UMAP plot was used to visualize the hub genes.

### Flow cytometry was used to detect and sort ILC subsets

Cardiac mononuclear cell suspension of MI/MIRI/sham mice was used to verify ILC subsets (ILCdc, ILC2a, ILC1, ILCt). Each group contained three samples, and 3-5 C57BL/6J mice were mixed into one sample. Cardiac mononuclear cells were obtained as described above. ILCdc was identified by the fluorescently labeled antibody Lin^-^CD45^+^CD127^+^CD74^+^, ILC2a was identified by the fluorescently labeled antibodies Lin^-^CD45^+^CD127^+^ST2^+^, ILC1 was identified by the fluorescently labeled antibodies Lin^-^CD45^+^CD127^+^NKP46^+^ and ILCt were identified by the fluorescently labeled antibodies Lin^-^CD45^+^CD127^+^CD2^+^. ILC subsets were sorted by the flow cell sorter Becklo-MoFlo-XDP, and 200 to 500 cells per sample were collected for subsequent qRT-PCR. In addition, we set FMO (Fluorescence Minus One) as control to improve the accuracy of the flow cytometry gating strategy when detecting cardiac mononuclear cells.

### qRT-PCR validation

Based on RT-PCR amplification methods, the sorted ILC subsets were reverse- transcribed to obtain cDNA. cDNAs were synthesized with a Single Cell Sequence Specific Amplification Kit (Vazyme, Nanjing, China). First, the amplification primers of different genes were mixed to make an Assay Pool (the final concentration of each primer was 0.1 μM), and then the reaction system was configured in the Nuclease-free centrifuge tube as follows: 2x Reaction Mix 2.5ul, 0.1 uM Assay Pool 0.5ul, RT/Taq enzyme 0.1ul, Nuclease-free ddH20 up to 5.0 μl. Single-cell ILC subsets were added to the reaction system, and then the following reaction was performed on the PCR instrument: 50° for 60 min and 1 cycle, 95° for 3 min and 1 cycle, 95° for 15 s, 60° for 15 min and 14 cycles. At the end of the reaction, 20 μl of Nuclease-free ddH2O (1:5 dilution) was added to each tube, centrifuged at 3000 rpm (1000 × g) for 2 min, and subsequent qPCR reactions were performed immediately.

The qRT-PCR reactions were carried out in the Bio-Rad CFX96 Real-time PCR Detection System, using AceQ^®^ qPCR SYBR Green Master Mix (Vazyme, Nanjing, China). The program was set as a two-step method, 95° for 10 s, 60° for 30 s and 40 cycles. The gene expression results were analyzed using the 2^−ΔΔCT method, and GAPDH was used as an endogenous control for mRNA expression.

### Statistical analysis

SAS was used for statistical analysis. For flow cytometry data analysis, two-tailed unpaired T-test was used to compare the differences between two groups, and one-way ANOVA was used to compare the differences between multiple groups. The data were expressed as mean ± sd, n = 3. *P*<0.05 indicated statistical significance.

## Results

### Clustering, visualization and identification of ILCs in mouse heart

**Figure 1:**
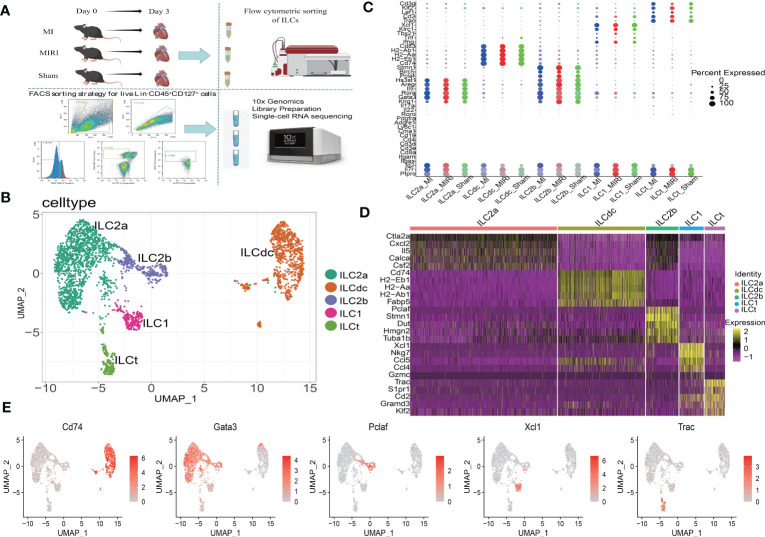
Identification of ILC clusters in mice ischemic myocardium by scRNA-seq. **(A)** Workflow of the experimental procedure: mice sham/MI/MIRI model preparation, cardiac mononuclear cell suspension extraction 3 days later, flow sorting for ILCs (Lin^-^CD45^+^CD127^+^) and 10X genomics scRNA sequencing. **(B)** Visualization of ILC clusters infiltrated in ischemic myocardium of mice by UMAP. **(C)** Dot plots represent the marker genes of ILC clusters in different disease groups; the size of the dots represents the percentage of marked gene expression in the different groups. **(D)** Heatmap shows the top 5 genes for each ILCs subcluster. **(E)** Representative molecular signatures of each ILC cluster are displayed by feature plots.

To elucidate the cellular constitutions of ILCs in mouse heart, we performed scRNA-seq using 10X technology (**Figure 1A**). Live heart ILCs (Lin^-^CD45^+^CD127^+^) from mice in the sham, MI and MIRI models were sorted by a fluorescence-activated cell sorter (FACS) (**Figures S1A–C**). The quality control of scRNA-seq is shown in **Figures S1D, E**. It is worth noting that the number of doublets identified in **Figure S1F** was very low and these cells were subsequently removed. Then, to further remove low-quality cells, cells were screened by setting the thresholds as the expression of Cd127 (Il7r) and Cd45 (Ptprc) and no expression of Cd3e, Cd3d, Cd4, Cd8a, Cd19, Cd11c(Itgax), Cd11b (Itgam), Cma1, Ly6c1, Adgre1 (F4/80) or Pdgfra. Finally, 1365, 962 and 495 cells were screened from the MI, MIRI and sham groups, respectively.

Unbiased clustering of the cells identified five clusters based on uniform manifold approximation and projection (UMAP) analyses (**Figure 1B**). We then annotated the clusters by using the FindAllMarker function to calculate marker genes for each cluster by using scType as a reference, and in combination with the ILC- specific marker genes reported in the literatures. Finally, five clusters, namely ILC1, ILC2a, ILC2b, ILCdc and ILCt, were obtained (**Figure 1B**). As shown in **Figure 1C**, Ptprc and Il7r were present in every cluster; at the same time, Cd4, Cd3d, Cd3e and Cd8a in T lymphoid cells, Cd19 in B cells, Itgam in myeloid cells, Itgax in dendritic cells, Ly6c1 in monocyte cells, Adgre1 in macrophages, Cma1 in mast cells and Pdgfra in fibroblasts were not expressed. Inhibitor of DNA-binding protein 2 (Id2), which is a crucial transcriptional regulator for ILC development, was present in every cluster. These results confirmed the successful purification of ILC subsets. Interestingly, the expression levels for typical ILC3 markers, including Rorc, Il17a and Il22, were very low. In clusters ILC2a and ILC2b, the expression levels of Klrg1, Gata3, Rora, Il5 and Areg were high. In addition, ILC2b highly expressed Stmn1, Birc5 and Pclaf, indicating that this cluster may be different from classical ILC2s. In cluster ILC1, Klrc1 and Ifng were highly expressed when compared to other clusters. Meanwhile, the expressions of other ILC1 markers, such as Tbx21and Tnf, were also high. DC markers, including Cd74, Cd83, H2-Ab1, H2-Aa and H2-Eb1, were highly expressed in the ILCdc cluster, indicating the potential DC-like roles this cluster may play. T-cell surface glycoprotein CD3 gamma chain (Cd3g) and lymphoid enhancer-binding factor 1 (Lef1), which are important during T cell development and activation, were highly expressed in the ILCt cluster.

In order to identify gene expression patterns in the ILCs subgroups, particularly for some novel genes, a heatmap using the top five genes in each cluster was performed as demonstrated in **Figure 1D**. All the differentially expressed genes (DEGs) are presented in **Table S1**. In addition, the expression of representative marker genes in the cell populations is shown in **Figure 1E**. We also checked the expression of ILCreg- related genes, such as Id3, Il1b and Bcl2a1b, and found that they were highly expressed in ILCdc (**Figures S1G–I**). Moreover, proliferation-related gene expression shown by feature plots, such as Mki67, Pcna and Top2a, were highly expressed in the ILC2b cluster (**Figures S1J–L**).

### Proportion changes, hdWGCNA analysis and enrichment analysis of ILCs in MI/MIRI/Sham

**Figure 2:**
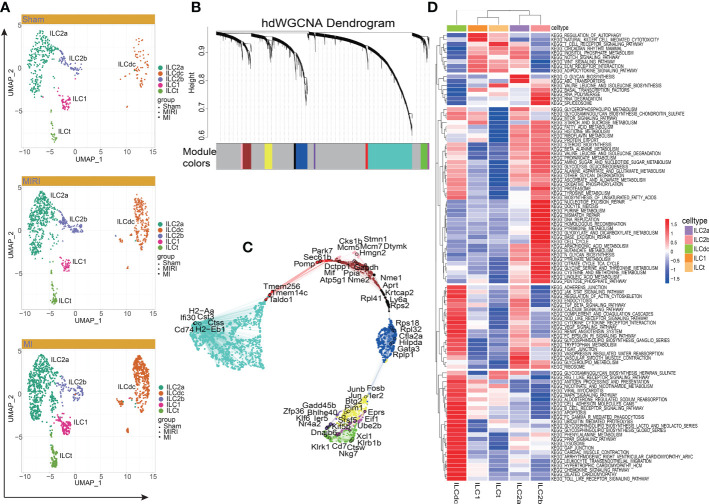
Cluster of modular genes, enrichment analysis and hub gene identification of ILC clusters. **(A)** UMAP visualizes the distribution of ILC clusters in different disease groups. **(B)** hdWGCNA dendrogram shows the different co-expression modules after network analysis. Each leaf represents a single gene, and the color indicates the co-expression module assignment. **(C)** The “ModuleUMAPPlot” function is used to plot the genes and their co-expression relationships. Each module displays the top 6 hub genes. **(D)** GSVA displays the KEGG enrichment pathway of ILC clusters. A deeper red gradient represents a higher degree of enrichment.

To investigate the differential changes of ILC subsets in the ischemic myocardium of mice, we visualized them with UMAP plots (**Figure 2A**) and calculated the number and proportion of ILC subsets in the mouse MI/MIRI/Sham group (**Figure S2A**). In general, cells from every group were present in every cluster. It ‘s worth noting that the ILCdc and ILC2a clusters accounted for a large number and percentage, meaning that they are important ILC subclusters.

Next, to predict hub genes and construct gene regulatory networks in each ILC cluster, hdWGCNA was used. As shown in **Figure 2B**, ten major gene modules and the related top ten genes in each module were identified (**Figure S2B**). Then, after comparing with DEGs in ILCs (**Table S1**), these gene modules were clustered into ILC clusters as shown in **Figure 1B** according to the genetic similarities (**Figure S2C**). GO-Biological Process and Molecular Function were then performed to predict ILC-related signal pathways (**Figures S2D, E**). ILC2a was involved in cytoplasmic translation, cotranslational protein targeting to membrane and SRP-dependent cotranslational protein targeting to membrane. Furthermore, ILC2a may have functions in large ribosomal subunit rRNA binding, ubiquitin-protein transferase inhibitor and ubiquitin ligase inhibitor activity. ILCdc was involved in the regulation of neutrophil activation and played a role in IgG binding, superoxide-generating NAD(P)H oxidase activity and oxidoreductase activity. ILC2b was involved in nuclear cell cycle DNA replication, double-strand break repair *via* break-induced replication and mitotic DNA replication. Additionally, ILC2b may play a role in DNA replication origin binding, single-stranded DNA helicase activity and 3’−5’ DNA helicase activity. The hub genes in each module are shown in **Figure 2C**. We specifically highlighted our hdWGCNA analysis for ILCdc, ILC2a and ILC2b. CD74 was predicted to be a crucial gene for ILCdc. Finally, the gene regulatory networks are demonstrated in **Figure S2F**. In general, the hub genes obtained here had high correlation with the aforementioned DEGs.

In order to explore the potential biological functions of ILC subsets, we used the “GSVA” R package for pathway enrichment analysis (**Figure 2D**). GSVA analysis showed that the ILC subgroups were involved in 186 signaling pathways (**Table S2**). ILC2a was mainly involved in O-glycan biosynthesis, ABC transporters, vascular smooth muscle contraction, Notch signaling pathway, glycerolipid metabolism and inositol phosphate metabolism. ILC2b was mainly involved in base excision repair, nucleotide excision repair, mismatch repair, DNA replication, RNA polymerase, RNA degradation, basal transcription factors, spliceosome, cell cycle, homologous recombination, purine metabolism and pyrimidine metabolism. ILC1 was mainly involved in the regulation of autophagy, natural-killer-cell-mediated cytotoxicity, adipocytokine signaling pathway and basal transcription factors. Additionally, ILCdc was involved in antigen processing and presentation, chemokine signaling pathways, Toll -like receptor signaling pathway, Jak STAT signaling pathway, MAPK signal pathway, PPAR signal pathway, leukocyte transendothelial migration, apoptosis, renin angiotensin system, cardiac muscle contraction, dilated cardiomyopathy and hypertrophic cardiomyopathy. ILCt was mainly involved in T -cell receptor signaling pathway, valine leucine and isoleucine biosynthesis, RIG1-like receptor signaling pathway, adipocytokine signaling pathway, WNT signaling pathway, ECM receptor interaction and regulation of autophagy. Through GSVA analysis, we can understand the different potential biological functions of ILC clusters better.

### Pseudotime trajectory analysis of ILCs

**Figure 3:**
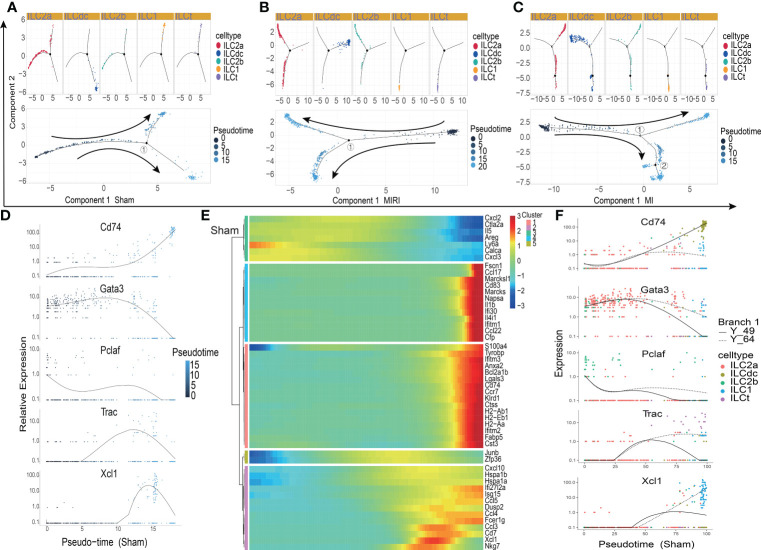
Pseudotime analysis of ILC clusters differentiation in different disease groups. **(A–C)** Distribution status of ILC clusters on pseudotime trajectory in the sham **(A)**, MIRI **(B)** and MI groups **(C)**. Cells on the tree are colored by cell type (upper panel) and pseudotime (lower panel) Each dot represents a cell and the coloring from dark to light represents the ordering of the pseudo-time; the darker the color gradient, the earlier the temporal development; the black dot represents the branch point with numbers and arrows represent the direction of differentiation. **(D)** The relative expression of typical marker genes, including Cd74, Gata3, Pclaf, Trac and Xcl1, in each ILC cluster during pseudotime. **(E)** Several gene cohorts that changed during the pseudotime transition. From left to right represents the pseudo-temporal order, and color gradients represent gene expression levels. **(F)** Pseudotime kinetics of Cd74, Gata3, Pclaf, Trac, and Xcl1 in the presence of two differentiation fates at branch point 1 in the sham group; Y_49 (solid line) represents cell fate 1, and Y_64 (dotted line) represents cell fate 2.

In order to explore the gene expression of ILCs subgroups in different disease conditions, we used Monocle2 software to conduct a pseudotime analysis. As showed in **Figures 3A–C**, the five ILC subclusters were ordered along a trajectory, and different states with branching points were identified for the sham, MIRI and MI groups. For ILCs at rest conditions (**Figure 3A**), ILC2a and ILC2b were found in the early stage of the trajectory, and ILCdc was found in the later stage of the trajectory; meanwhile, ILC1 and ILCt were also found in the later stage but not in the same branch as ILCdc. Interesting, the ILC clusters in ischemic heart showed different trajectories. ILCdc was found in the early stage of the trajectory for both the MIRI and MI group, and ILC2a and ILC2b were found within the both two branches of the later stage of the trajectory; meanwhile, ILC1 and ILCt were within the same branch of the later stage (**Figures 3B, C**). Notable, there were two branch points for MI but only one point for both the sham and MIRI group. Marker gene expressions of different ILC clusters were further demonstrated and confirmed the cell distributions along the pseudotime trajectory (**Figure 3D**, **Figures S3A, B**). For example, ILCdc showed a rising trend in the sham group and a downward trajectory in the MI and MIRI groups, but ILC2a and ILC2b showed an opposite pattern.

Further analysis identified several gene cohorts that changed during the pseudotime transition (**Figure 3E**, **Figures S3C, D**). Notably, the expression levels of ILC2s-related genes, such as Il5 and Areg, decreased, while ILCdc-related genes, such as CD74, H2-Aa and Cst3, increased during pseudotime. Gene expression patterns were also identified between the two transition states (**Figure 3F**, **Figures S3E, F**). Analysis of branch point one for the sham group discovered the upregulation of ILCdc-related genes such as CD74, H2-Aa and Cst3 at cell fate 1 (**Figure S2G**). Upregulation of ILC1-related genes, such as Xcl1 and Gzmb, was revealed at cell fate 2. A similar analysis for the MIRI and MI groups was performed and is demonstrated in **Figures S3G, H**. However, interestingly, the expressions of ILCdc-related genes, such as CD74, H2-Aa and Cst3, were high at the pre-branch point and then decreased in both cell fate 1 and cell fate 2 in the MI group.

### The transcription factors that regulate ILC subsets

To further assess the mechanisms underlying the heterogeneity of ILCs infiltrating the ischemic myocardium, we used SCENIC software to predict and analyze the transcription factors that regulate ILC subsets. SCENIC allowed the identification of TF-centered gene co-expression networks. As expected, a set of TFs implicated in heart ILCs exhibited different expression patterns among the sham, MI and MIRI groups and also among different ILC clusters. We identified previously known transcription factors that regulate ILC subsets, and also identified some novel transcription factors involved in regulating the fates of ILCs subsets.

**Figure 4:**
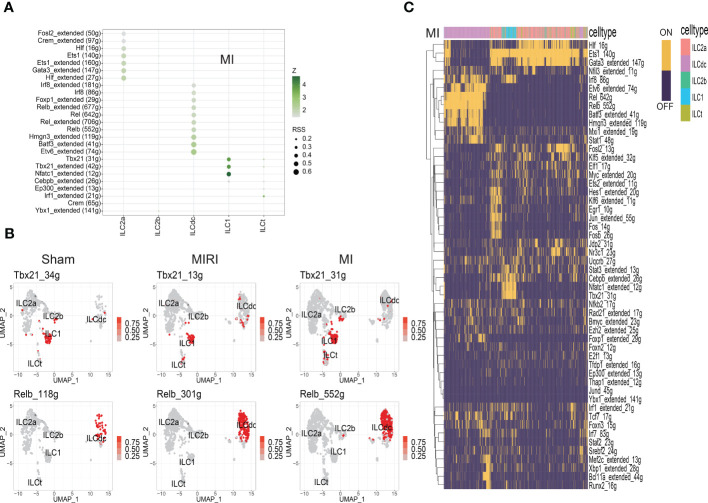
Analysis of ILC clusters TF in ischemic myocardium by SCENIC. **(A)** RSS (Regulon Specificity Score) was used to identify ILC cluster- specific regulons in the MI group. The color depth represents the z-score value, and the size of the point represents the RSS score. **(B)** The AUC matrix was converted to binary matrix, and Featureplot displays the activated transcription factors in the Sham/MIRI/MI groups, respectively. **(C)** Heatmap shows the binary converted regulators activated in the MI group; ON represents activated transcription factors and OFF represents non-activated transcription factors.

TFs with high regulon specificity scores for every cluster in different groups a re demonstrated in **Figure 4A** and **Figures S4A, B**. Tbx21 was predominantly expressed in ILC1s in all three groups in our results as a previous well-known TF for ILC1s (**Figure 4B**). Furthermore, our results revealed upregulated expression of Irf8 in the sham group and increased activity of Nfatc1 in the MI group for ILC1. For the ILCt cluster, Irf1 was the TF with high regulon specificity score in both the sham and MI groups; meanwhile, Lef1 was the special TF in the MIRI group. Interestingly, for the ILC2b cluster, Tfdb1 was the potential TF in the sham group, but there was no special TF for the MI and MIRI groups.

As shown in **Figure 4A** and **Figures S4A, B**, ILC2a and ILCdc had more specific regulons than other clusters, indicating the higher cell activities they may have. ILC2a showed high regulon activity of GATA3 in all three groups as expected, and additional increased TF regulon signatures included Rora/Nfkb/Ets/Fosb/Junb/Jund for the sham group, Hif/Crem/Fosl2 for the MI group and Ets1/Crem for the MIRI group. Furthermore, ILCdc had high regulon activity of Rel/Irf5/Tcf4/Bcl11a/Bach1/Spib, Etv6/Batf3/Hmgn3/Rel/Foxp1/Irf8 and Irf5/Relb/Batf3 for the sham, MI and MIRI groups, respectively. We specifically highlighted our SCENIC analysis for ILCdc and found that Relb was predominantly expressed in the ILCdc cluster (**Figure 4B**). Additionally, we also displayed the heatmaps of different TFs expressions in ILC clusters for different groups in **Figure 4C** and **Figures S4C, D**.

### Cell communications among heart ILC clusters in different conditions

**Figure 5:**
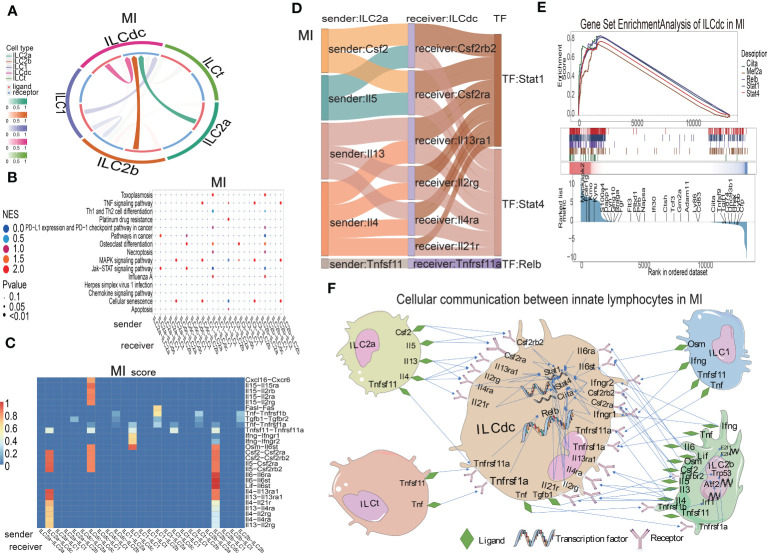
Cellular communications among ILCs clusters in mouse MI myocardium. **(A)** Circos plot shows the ligand–receptor interactions across and within ILC clusters in the MI group. **(B)** Bubble plot shows the enrichment pathway of paired ligand–receptor data set between signal sender and receiver in the MI group. In the X-axis, the former represents the signal sender, the latter represents the signal receiver and the Y-axis represents the enriched signaling pathways. The higher the NES score, the closer the enrichment of the pathway. **(C)** Heatmap shows the ligand–receptor pairs involved between the signal sender and receiver cells in the MI group. On the X-axis, the former represents the signal sender, the latter represents the signal receiver, and the Y-axis represents the ligand–receptor pair. The higher the score, the closer the cell communication. **(D)** Sankey plot displays the cell communication between ILC2a and ILCdc and downstream TFs in the MI group; the former represents the signal sender, the latter represents the signal receiver, and the TF represents the transcription factor that is activated in the signal receiver. **(E)** GSEA analysis for TF regulation of downstream genes in MI. **(F)** Displaying the cellular communication pattern among ILCdc and other ILC clusters through the ligand–receptor–TF mode.

ILCs have been found to play roles though communications with other cells in several diseases. In order to study the interaction relationships among the ILC subclusters, we used the CellCall toolkit to uncover the communication networks in the sham, MIRI and MI groups. As shown in **Figure 5A** and **Figures S5A, B**, ILCdc was the prominent receiver of signals from other cells in the sham, MIRI and MI groups, suggesting the importance of ILCdc in ischemic heart. Hence, we focused on ILCdc in the following analysis. Pathway activity analysis showed that the intercellular signal from ILC2b to ILCdc was mainly enriched in the TNF signaling pathway in the sham group, JAK-STAT signaling pathway in the MIRI group, and necroptosis in the MI group (**Figure 5B**, **Figures S5C, D**). In addition, the intercellular signal from ILC2a to ILCdc was mainly enriched in the JAK-STAT signal pathway for the MIRI and MI groups. Further ligand–receptor interaction heatmaps demonstrated that ILC2b interacted with ILCdc through Vegfb-Kdr, Vegfc-Kdr, Flt3l-Flt3, Tnfsf11-Tnfrsf11a and Ccl4-Ccr1 in the sham group, and through Csf2-Csf2ra/Csf2rb2, Il5-Csf2ra/Csf2rb2, Lif-Il6st, Il4-Il13ra1/Il4ra/Il21r, Il6-Il6st, Il13-Il13ra1/Il2rg/Il4ra and Tnfsf11-Tnfrsf11a in the MIRI and MI groups (**Figure 5C**, **Figures S5E, F**). Additionally, ILC2a interacted with ILCdc through Flt3l-Flt3 and Tnfsf11-Tnfrsf11a in the sham group, but through Csf2-Csf2ra/Csf2rb2, Il5-Csf2ra/Csf2rb2 and Il13-Il13ra1/Il2rg/Il4ra in the MIRI and MI groups.

Then, Sankey diagrams were used to demonstrate the ligand–receptor–transcription factor regulatory axis (L-R-TF) among ILCs in the heart. For example, in the MI group, ILC2a may secrete Il5, which is received by Csf2ra and Csf2rb2 in ILCdc, and then transcription factor Stat4 is activated (Il5-Csf2ra/Csf2rb2-Stat4) (**Figure 5D**). It is worth noting that this conclusion was predicted by cell communication “cellcall”, which needs to be further verified by experiments. Tnfsf11-Tnfrsf1la-Relb, Il4-Il13ra1/Il2rg/Il4ra/Il21r-Stat4, Il13-Il13ra1/Il2rg/Il4ra-Stat1 and Csf2-Csf2rb2/Csf2ra-Stat1 were the other L-R-TF axis for ILC2a and ILCdc in the MI group. The L-R-TF axis between ILC2a and ILCdc in the sham and MIRI groups are also shown in **Figures S5G, H**. Interestingly, the messages derived from ILCdc only sent to ILCdc itself and ILC2b (**Figure 5A**, **Figures S5A, B**). Sankey diagrams for ILCdc-ILC2b and ILCdc-ILCdc L-R-TF axis are shown in **Figures S5I, J**. The ligands for ILCdc-ILC2b connection were TNF and Tgfb1. In addition, ILCdc may have an autocrine effect on itself *via* several ligands, such as CXCL16. As shown in **Figures S5K, L**, ILCdc specially expressed CXCL16 and moderately expressed Tgfb1. Furthermore, to demonstrate the regulation of downstream genes by intracellular transcription factors for ILCdc, we used GSEA to display the set of genes downstream of transcription factor regulation (**Figure 5E**). Finally, the cellular communication pattern among ILCdc and other ILCs in MI is shown in **Figure 5F**.

### The heterogeneities and transcriptional features of ILCdc and ILC2a

**Figure 6:**
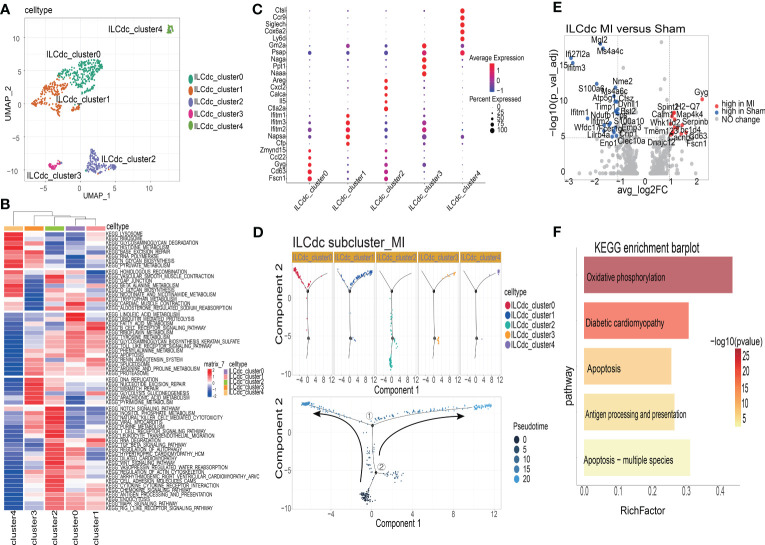
The heterogeneities and transcriptional features of ILCdc. **(A)** Visualization of ILCdc sub-clusters infiltrated in ischemic m yocardium of mice by UMAP. **(B)** GSVA displays ILCdc sub-clusters in KEGG enrichment pathway. **(C)** Dot plot displays the marker genes of ILCdc sub-clusters. **(D)** Distribution statuses of ILCdc subclusters on pseudotime trajectory in the MI group. **(E)** Volcano plot shows the DEGs of ILCdc between the MI group and sham group. **(F)** KEGG enrichment analysis of ILCdc DEGs between the MI and sham group.

To analyze the heterogeneity of ILCdc further, ILCdc was subjected to unsupervised clustering by using UMAP. Five subclusters were revealed with unique signature genes, named ILCdc clusters 0-4 (**Figure 6A**). ILCdc cluster 0 and cluster 1 were found to be the predominant clusters. In addition, GSVA showed the pathways that ILCdc clusters may be involved in (**Figure 6B**). Next, the unique signature genes of each ILCdc cluster were demonstrated in **Figure 6C**. Fscn1, CD63, Gyg, Ccl22 and Zmynd15 were the top five genes for ILCdc cluster 0, and Cfp, Napsa, Ifitm1, Ifitm2 and Ifitm3 were the top five genes for ILCdc cluster 1. Pseudotime trajectory analysis of ILCdc clusters was then performed to explore the development stages of ILCdc subgroups in the MI group. As shown in **Figure 6D**, ILCdc cluster 2 was found to be in the earliest stage. In addition, ILCdc clusters 0 and 1 were found to be in the late stage but in different cell fate directions. Further analysis demonstrated the gene cohorts that changed during the pseudotime transition for the ILCdc cluster in the MI group (**Figure S6A**). Gene expression patterns were also identified between the two transition states (**Figure S6B**). Analysis of branch point one for the MI group discovered the downregulation of ILCdc cluster 0 genes, such as CD63 and Gyg. Upregulation of ILCdc cluster 1-related genes, such as Ifitm1 and Ifitm3, were revealed at cell state 2.

To better reveal the transcriptional characteristics of ILCdc, several analysis methods were used here. Firstly, the volcano plot in **Figure 6E** shows the DEGs for ILCdc in the sham and MI groups (whole DEGs are provided in **Table S3**). The expression of Gyg, H2-Q7, Map4k4, Serpinb6b, Spint2, Calm1, Wnk1 and Cd63 were more highly expressed in the MI group, while that of Mgl2, Ms4a4c, Ifi27l2a and Ifitm3 were more highly expressed in the sham group for ILCdc. Interestingly, under the same threshold, there were no DEGs between the sham and MIRI groups. Then, KEGG enrichment analysis was performed using the DEGs acquired previously. **Figure 6F** shows that ILCdc was involved in oxidative phosphorylation and apoptosis in the MI group.

Next, we compared the gene expression patterns of ILCdc between the sham and MI groups by using pseudotime trajectory analysis. As shown in **Figure S6C**, ILCdc in the sham groups was predicted to be in the earlier stage; meanwhile, ILCdc in the MI group was predicted to be in the later stage with two different cell fates. Gene cohorts that changed during the pseudotime transition and gene expression patterns between the two transition states are also shown in **Figures S6D, E**.

In a similar manner, we explored the transcriptional features of ILC2a. As shown in **Figure S7A**, ILC2a was divided into four subclusters, named ILC2a clusters 0-3. The cell number and proportion of ILC2a subclusters are shown in **Figures S7B, C**, indicating that ILC2a clusters 0-2 shared a similar expression level. The top five genes for ILC2a cluster 2 were Ramp3, Gata3, Isy1, Il5 and Il13, suggesting that this subcluster was the classical ILC2 (**Figure S7D**). Furthermore, the top five genes for ILC2a cluster 0 were Pkm, Ly6a, AA467197, Nme1 and Mdh1. Additionally, for ILC2a cluster 1, the top five genes were Zfp36, Fos, CD69, Atf3 and Hspa1b. The pseudotime analysis suggested that ILC2a clusters 0 and 3 were in the earliest stage, while clusters 0 and 2 were in the middle stage and cluster 1 was in the later stage (**Figure S7E**). Gene cohorts that changed during the pseudotime transition and gene expression patterns between the two transition states were also identified in **Figures S7F, G**.

### Identification of ILC subsets by flow cytometry

**Figure 7:**
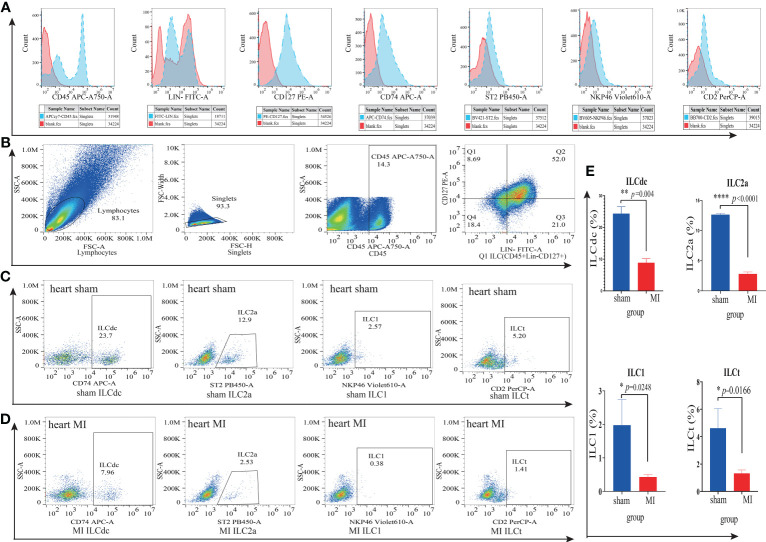
ILC subsets in cardiac mononuclear cells in the MI and sham groups were identified by flow cytometry. **(A)** The blank control fluorescence channel and single- antibody- labeled fluorescence channel are shown by histogram. Red color represents the blank control fluorescence channel and blue color represents the fluorescence channels of CD45-APC-A750, Lin-FITC, CD127-PE, CD74-APC, ST2-PB450, NKP46-Violet610 and CD2-Percp, respectively. The X-axis represents the value of the fluorescence channel, and the Y-axis represents the number of cells. **(B)** Flow cytometric gating strategy of ILC(Lin^-^CD45^+^CD127^+^) in cardiac mononuclear cells. Q1 represents ILC(Lin^-^CD45^+^CD127^+^). **(C, D)** Identification of ILCdc(Lin^-^CD45^+^CD127^+^CD74^+^), LC2a(Lin^-^CD45^+^CD127^+^ ST2^+^), ILC1(Lin^-^CD45^+^CD127^+^NKP46^+^) and ILCt(Lin^-^CD45^+^CD127^+^CD2^+^) in cardiac mononuclear cells by flow cytometry represents the proportions of ILCdc, ILC2a, ILC1, and ILCt in sham and MI groups, respectively. **(E)** Comparison of ILCdc, ILC2a, ILC1, and ILCt between sham and MI groups by two-tailed unpaired T -test, respectively, n=3, 3-5 mice per sample. * represents a significant difference (p<0.05), ** represents a significant difference (p<0.01), **** represents a significant difference (p<0.0001), ns represents no significant difference.

To further verify the existence of ILC subsets, we used flow cytometry to identify ILCdc (Lin^-^CD45^+^CD127^+^CD74^+^), ILC2a (Lin^-^CD45^+^CD127^+^ST2^+^), ILC1(Lin^-^CD45^+^CD127^+^NKP46^+^) and ILCt (Lin^-^CD45^+^CD127^+^CD2^+^). Heart and spleen mononuclear cells in the MI/MIRI/sham groups were isolated and FACS was performed. Here, we plot the flow cytometric gating strategy for ILC and list the histograms of fluorescence channels for single antibodies versus blank controls separately (**Figures 7A, B**). In addition, we set FMO (Fluorescence Minus One) as control to improve the accuracy of the flow- cytometry gating strategy when detecting ILCdc (**Figure S8B**), and the presence of ILCdc was confirmed in both the heart and spleen (**Figures S8C, S10**). In the heart tissue, compared with the sham group, the ILCdc in the MI group decreased significantly (*P*=0.004) (**Figures 7C–E**). When compared with the sham group, there was no significant difference in the MIRI group (*P*=0.3575) (**Figures S9C–E**).

For other ILCs clusters, when compared with sham group, the proportion of ILC2a decreased significantly both in the MI group (P<0.0001, **Figures 7C–E**) and in the MIRI group (P=0.0009, **Figures S9C–E**).

And the proportion of ILC1 in the MI group decreased significantly (*P*=0.0248) (**Figures 7C–E**) but showed no significant difference in the MIRI group (*P*=0.50) (**Figures S9C–E**). As for ILCt, the proportion of ILCt in the MI group decreased significantly (*P*=0.017) (**Figures 7C–E**) when compared with the sham group, and there was no significant difference in the MIRI group (*P*=0.49) (**Figures S9C–E**).

In the spleen, ILCdc was decreased significantly in the MI group when compared with the sham group (*p*=0.037), but there was no significant difference between the MIRI group and sham group (*p*=0.96) (**Figures S10C, D**).

### Marker genes of ILC subsets were verified by qRT-PCR

**Figure 8:**
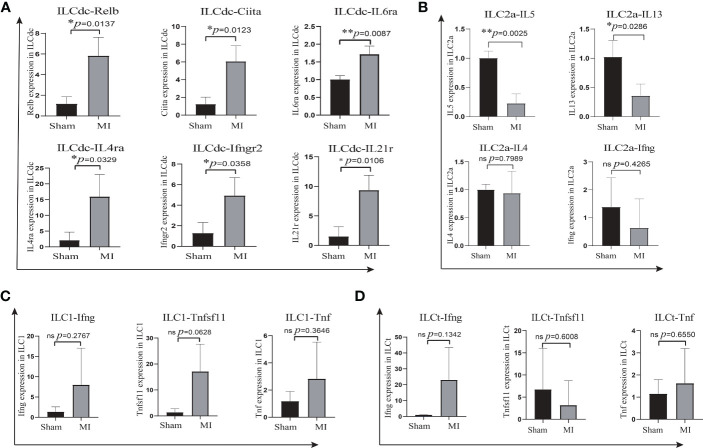
Marker genes in ILC subsets were verified by qRT-PCR. **(A)** Comparison of Relb, Ciita, IL6ra, IL4ra, Ifngr2 and IL21r expression in ILCdc between sham and MI groups by two-tailed unpaired T- test, n=3, 500 cells per sample. **(B)** Comparison of IL5, IL13, IL4 and Ifng expression in ILC2a between sham and MI groups by two-tailed unpaired T test, n=3, 500 cells per sample. **(C, D)** Comparison of Ifng, Tnfsf11 and Tnf expression in ILC1 and ILCt between sham and MI groups by two-tailed unpaired T test, n=3, 200 cells per sample. * represents a significant difference (p<0.05), ** represents a significant difference (p<0.01), ns represents no significant difference.

To further validate the marker genes of ILC subsets in cardiac mononuclear cells, we sorted ILC subsets (ILCdc, ILC2a, ILC1, ILCt) using flow cytometry and performed qRT-PCR. The results showed that the expressions of Relb, Ciita, IL6ra, IL4ra, Ifngr2 and IL21r in ILCdc were significantly higher in the MI group than those in the sham group (**Figure 8A**). The expressions of IL5 and IL13 in ILC2a were significantly lower in the MI group than those in the sham group (**Figure 8B**). However, the expression of IL4 and Ifng in ILC2a was not significantly different in the MI group when compared with the sham group (**Figure 8B**). The expression of Ifng, Tnfsf11 and Tnf in ILC1 showed an upward trend in the MI group, but there was no statistically significant difference when compared with the sham group (**Figure 8C**). The expression of Ifng, Tnfsf11, and Tnf in ILCt was not significantly different between the MI and sham groups (**Figure 8D**).

## Discussion

Here, we report the cellular complexity of ILC subsets in the ischemic myocardia of normal, MI and MIRI mice. To our knowledge, this is the first paper to dissect the cardiac ILCs and their communication networks. Five ILC subsets were identified, including ILC1, ILC2a, ILC2b, ILCdc and ILCt. All five ILCs clusters were Lin^-^ and highly expressed ILCs canonical genes, such as CD127, CD45 and Id2, meeting the dictionary definition of ILCs.

ILCdc, a separate group in terms of spatial distance in UMAP graph, is an important subset of ILCs which retain both ILCs and dendritic cell markers, such as CD74, H2-Eb1, H2-AA, H2-Ab1, CCR7 and CST3. ILCdc lacked the expression of transcription factors GATA3, TBX21 and RORγt, indicating that it was different from ILC1s, ILC2s and ILC3s. We carefully evaluated the possibility of doublets, which are serious confounders in scRNA-seq analysis. Doublets are artifactual libraries of two cells. We filtered the doublets in the upstream data processing and double-checked the doublets in the quality control step by using DoubletFinder package, finally eliminating 45 potential double cells. Furthermore, our FACS showed that ILCdc cells were expressed in the heart and spleen. Compared with the sham group, ILCdc in the MI and MIRI groups showed decreasing trends in the heart and spleen. Considering that in the acute phase of myocardial ischemia, a severe inflammatory response is activated, and a variety of immune cells play different functions, some immune cells undergo necrosis and apoptosis in order to maintain tissue homeostasis and some cells play a pro-inflammatory role, it is reasonable to assume that ILCdc may play roles in MI/MIRI by functioning as an immune defense cell. However, more research is needed to clarify this.

It is widely believed that ILCs are the innate counterparts of T cells as they have similar functions and secretion profiles, with ILC1 mirroring CD4 T helper Th1, ILC2 mirroring Th2 and ILC3 mirroring Th17 subsets (36). Is ILCdc a novel ILC subset and a counterpart of dendritic cells? Gury-BenAri M et al. characterized the spectrum of small intestinal ILCs and divided them into 15 subgroups (37). They found that a subgroup of ILCs highly expressed Cd74, H2-AA, Gpx1, Cx3cl1 and Il17f and classified it as ILC3e. The transcriptional pattern was somehow similar to our ILCdc cluster. However, more details and function assays are needed to further compare these two cells. Later, Zhou et al. found a Zbtb46^+^ILC3s subgroup which not only expressed RORγt, a transcription factor of ILC3s subgroup, but also expressed Zbtb46, a transcription factor specific for dendritic cells (38). The authors found that the Zbtb46^+^ILC3s subgroup inhibited intestinal inflammation by secreting IL22. Zbtb46 was proposed to be a transcription factor shared between dendritic cells and ILC3s. Moreover, recent studies have identified cells that share some characteristics of ILC3 and DC (39–41). Akagbosu et al. identified a novel antigen presenting cell- imparted Treg-dependent tolerance to gut microbiota (42). The finding of overlapping markers between this new cell and DC helped resolve the relationship between RORγt^+^ dendritic cells and ILC3s. Furthermore, Tobias K. Vogt’s research found that Bone marrow–derived dendritic cells (BMDCs) expressed IL-7 receptor (IL-7R) and survived significantly longer in the presence of IL-7. Migratory DCs (migDCs) isolated from lymph nodes also expressed IL-7R, and they proposed that a large fraction of Conventional DCs (cDCs) and plasmacytoid DCs (pDCs) derived from CLPs and shared not only the lymphoid origin but also the IL-7 requirement with lymphocyte precursors (43). Besides, Joannah R. Fergusson analyzed the “extra-thymic auto-immune regulator (AIRE) expressing cells” (eTACs) using combinations of flow cytometry and single cell RNA-sequencing, and they identified AIRE^+^ cells as CD127^+^dendritic cells in human tonsils (44). Therefore, it is reasonable to believe that there is a kind of mirror cell similar to dendritic cells in the ILC subpopulation.

Furthermore, our GSVA results showed that ILCdc was involved in cellular antigen presentation function, similar to that of dendritic cells. Recently, several papers have reported the antigen-presenting properties of ILCs. Björklund et al. demonstrated the presence of a distinct ILC3 population in human tonsils which expressed CD74 and CTSS, implying the capacity to present antigens (45). Rao et al. reported that intestinal and peripheral blood ILCs acquired antigen-presenting characteristics triggered by IL-1β and IL-18 (46). In addition, our hdWGCNA analysis showed that CD74 was an important hub gene for ILCdc. All of this indicates the antigen-presenting potential of ILCdc.

Pseudotime algorithms were used to extract latent temporal information of cell clusters in different situations. Our pseudotime analysis demonstrated that ILCdc changed from “late stage” in the sham group to “early stage” in the MIRI and MI groups. In addition, FACS results manifested that the proportion of ILCdc in the heart decreased in the MI group, and similarly, the proportion of peripheral ILCdc also decreased, suggesting ILCdc may function as a fighter in the first line against ischemia- related inflammation and immune reaction. It was reported that ILCs were tissue- resident under homeostatic conditions. Different pathogenic stimuli, such as ischemia and inflammation, may affect the phenotype and functions of ILCs. Baran et al. found that the cardiac ILC population was mainly undifferentiated ILC2 phenotype at rest conditions. However, during ischemia and myocarditis, cardiac ILCs could differentiate into ILC2s, but not ILC1s and ILC3s (47). Moreover, when cells are subjected to inflammation or ischemia, they make fate choices and may appear as branches in single-cell pseudotime trajectories. Our results demonstrated the gene expression patterns of ILC clusters in the sham, MI and MIRI groups. These crucial genes, to a certain degree, may uncover the underlying mechanisms of different ILC development, plasticity and function in ischemic heart, and may provide potential drug targets in future.

Recently, Bat et al. reviewed the plasticity of ILCs (48). The conversions of ILC2s to ILC1s, ILC3s to ILC1s, ILC1s to ILC3s and ILC2s to ILC3s were well summarized. Interestingly, IL-12-producing dendritic cells may be involved in the conversion of IL-22-producing ILC3s into IFNγ-producing ILC1s (49). Additionally, it is noteworthy that there is no direct evidence for the conversions of ILC1s and ILC3s to ILC2s. Another example of ILC plasticity was reflected in ILC2s itself. Inflammatory ILC2s (iILC2s), which were IL-25- responding IL-17RB^+^ ILC2s and circulating, expressing both RORγt and GATA3, could produce both IL-13 and IL-17. Natural ILC2s (nILC2s), which were IL-33 -responding, were tissue resident. Previous data show that iILC2s can convert into nILC2s *in vivo* and *in vitro* and vice versa (50). Whether the ILCs clusters identified here can convert into each other needs further verification.

Our SCENIC analysis suggested that Relb was a highly activated TF for ILCdc. Relb is involved in the canonical and noncanonical NF-κB pathway (51, 52). In addition, Relb was reported to regulate DC, and its absence can promote airway inflammation (53). Zhang et al. reported that Relb could selectively restrain ILC2s, but not ILC1s or ILC3s (54). Furthermore, they found that Relb could suppress type 2 immune pathology *in vivo* by suppressing Bcl11b activity, which was critical in maintaining ILC2s identity. Our results here suggest that ILCdc may play roles in MI and MIRI through Relb. Future gene knockout mice studies may help further illuminate this.

ILCs have been found to play roles in many diseases through cell communication (55, 56). The CellCall package was used to draw a cellular communication map among ILC clusters through ligand–receptor–transcription factor (L-R-T) mode, which may help interpret how a cell listens and talks to another cell. Our results showed that ILCdc received messages from ILC2a, ILC2b, ILC1, ILCt and ILCdc; however, the messages derived from ILCdc were only sent to ILCdc itself and ILC2b. Tnfsf11-Tnfrsf1la-Relb was the pathway for ILCdc–ILCdc communication and the ligands for ILCdc–ILC2b connection were TNF and Tgfb1. It is well known that TNF may be produced by ILC1s. A recent study found that Tnfsf11 was able to induce IL-5 and IL-13 in ILC2s *via* Tnfsf11a in chronic rhinosinusitis with nasal polyps (57). In addition, Momiuchi et al. found that ILC2s in bone marrow could regulate osteoclastogenesis in a reciprocal manner through Tnfsf11 (58). Furthermore, Bando et al. reported that Tnfsf11 could suppress the abundance and effector cytokine production in intestinal CCR6^+^ ILC3s through ILC3-ILC3 interactions (59). In addition, ILCdc may have an autocrine effect on itself *via* CXCL16. Our data show that CXCL16 was highly expressed in the ILCdc cluster. Li et al. reported that CXCL6 was involved in ILC2 migration (60). Furthermore, Takayama et al. delineated a critical CXCL16–CXCR6 axis (DC-ILC3s crosstalk) that regulated intestinal ILC3s diversity and function (61). Hence, this autocrine effect may be involved in ILCdc migration in MI and MIRI conditions. Moreover, O’Connor reported a unique ILC population in human tonsils, termed follicular regulatory ILCs (ILCFR), which was uniquely identified by CD74 and ID3 expression (62). The authors predicted the role for ILCFR in regulating GC-Tfh-GC-B cell interactions. Interesting, besides expressing CD74, our ILCdc also moderately expressed characteristic genes similar to ILCreg, such as ID3, BCL2A1B and IL1B. This predicts that ILCdc may play a regulatory role in MI and MIRI. Future exploration of comparing our ILCdc with ILCFR significant, and may yield key insights into the connection between ILCs and DC.

Further analysis indicates that ILCdc can divide into five clusters. The marker genes for ILCdc cluster 0 include Fscn1, CD63, Gyg, Ccl22 and Zmynd15. Yamakita et al. reported that Fscn1 could promote cell migration of mature DCs (63). Ccl22 was reported to promote Tregs-DCs communication to control immunity (64). Interestingly, the marker genes for ILCdc cluster 2 include Areg, IL-5, Cxcl2, Calca and Ctla2a, suggesting the communication with ILC2s. Additionally, ILCdc KEGG enrichment analysis showed that MI ILCdc was involved in apoptosis, oxidative phosphorylation and diabetic cardiomyopathy when compared with the sham group, indicating that ILCdc may play important roles in MI. However, further evidence should be provided.

We also displayed the transcriptional characteristic of other ILC subclusters. Our study observed an increase in the number of total ILCs in mice, and ILC1 showed a decreasing trend; meanwhile, ILC2 showed a decreasing trend at 3 days after operation. In addition, our results showed that ILC2 was the most prevalent subset. All of these factors were consistent with previous research (14). ILC2s clusters included ILC2a and ILC2b. Both ILC2a and ILC2b highly expressed Gata3, Rorα, Il5, Areg and Klrg1. Compared with ILC2a, ILC2b particularly expressed Stmn1, Birc5 and Pclaf, all of which are cell-cycle-associated genes. We also studied other cell proliferation-related genes, MKI67, TOP2A and PCNA, and found that ILC2b expressions were again high. All of these factors indicate that I ILC2b exhibited a proliferative state 3 days after MI in mice.

Moreover, ILCt cells were also identified as a new subcluster of ILCs here, which highly expressed the genes LEF1, Trac, S1PR1, CD2, Gramd3, KLF2 and Cd3g. As we know, LEF1 is an important transcription factor for T -cell development, Trac is a TCR-related gene and CD2 and CD3G are signature genes of T cells. Hence, we hypothesized that ILCt may function in parallel with T cells. In fact, ILCs are regarded as the innate counterparts of Th1, Th2 and Th17 cells, but without surface antigen-specific T- cell receptor (TCR) expression (49). However, recent studies have found that NK cells show TCR recombination activity and rearrangement (65, 66). Furthermore, Mazzurana et al. found TCR-δ rearrangements in ILC1 subsets (67). Additionally, Shin et al. also found that lung ILC2s exhibited TCR rearrangements (68). It would be very meaningful to determine if ILCt is involved in TCR rearrangement through further assays, such as TCR sequencing, in future (69). Recently, CD4/8 double negative (DN) T cells were reported with characters that were CD3 positive but lack both CD4 and CD8 coreceptors, and could express either TCRβ or TCR gamma and delta (γδ) but did not express natural killer (NK) T cell markers (70). All these features are indeed very similar to ILCt. But importantly, ILCt does not express CD3, which is an important marker for T cells. More studies are needed in future to compare these two cells. Additionally, the relationship and communication between ILCs and T cells will be revealed more clearly in future as technology evolves.

Although this is the first study to describe ILCs transcriptional characteristics in mouse ischemic myocardial by single-cell sequencing, our study also has several limitations. First, when we sequenced single-cell RNA by flow sorting ILCs, only one sample from each group was used. Second, more western blot experiments and *in vivo* experiments are needed, to further explore the functions of the new ILC subsets. By combining single-cell sequencing results with further studies, the role of ILC subsets in ischemic myocardium may be revealed.

Together, we delineated novel subsets of ILCs infiltrating mouse ischemic myocardium by single-cell sequencing, and provided new evidence for exploring the heterogeneity and plasticity of ILCs. We believe that this study will help reveal the specific mechanism of ILC subsets in ischemic myocardium.

## Data availability statement

The datasets presented in this study can be found in online repositories. The names of the repository/repositories and accession number(s) can be found in the article/**Supplementary Material**.

## Ethics statement

The animal study was reviewed and approved by Animal Care and Use Committee of Union Hospital, Huazhong University of Science and Technology, China.

## Author contributions

Conceptualization, DL and JL. Methodology, SJ, YZ, WY, BW,SL. Software, JL, SJ and JZ. Data curation, YZ, BL, and SH. Original draft preparation SJ and SH. Review and editing, BL, YZ and JL. All authors contributed to the article and approved the submitted version.
