# Supplementary material for: Single-cell landscape dissecting the transcription and heterogeneity of innate lymphoid cells in ischemic heart
Source: Front Immunol. 2023 May 9;14:1129007. doi: 10.3389/fimmu.2023.1129007 (PMC10203554; doi:10.3389/fimmu.2023.1129007)
Supplement: Supplementary file 1 [file DataSheet_1.pdf]

Abbreviations

ILCs: innate lymphoid cells; MI: myocardial infarction; MIRI: myocardial ischemia-reperfusion injury; scRNA-seq: single-cell RNA sequencing; PCI: percutaneous coronary intervention; NK: natural killer cells; T-bet: T box transcript factor; RORα: retinoic acid receptor-related orphan receptor-α； GATA3: GATA binding protein 3; LTi: lymphoid tissue inducer; STEMI:ST-segment elevation myocardial infarction; IRI: ischemia-reperfusion injury; FACS: fluorescence-activated cell sorter; UMAP: uniform manifold approximation and projection; Id2: Inhibitor of DNA-binding protein 2; DCs: dendritic cells; Lef1: lymphoid enhancer-binding factor 1; DEGs: different expression genes; GSVA:Gene Set Variation Analysis; hdWGCNA: high dimensional weighted gene co-expression network analysis; SCENIC: single-cell regulatory network inference and clustering; GSEA: Gene Set EnrichmentAnalysis; KEGG: Kyoto Encyclopedia of Genes and Genomes; TFs: Transcription factors; L-R-T: ligand-receptor-transcription factor; ILCFR: follicular regulatory ILCs; LAD: left anterior descending coronary artery; PCA: principal component analysis; SNN: Shared nearest neighbor algorithm; KNN: k-Nearest Neighbors algorithm.

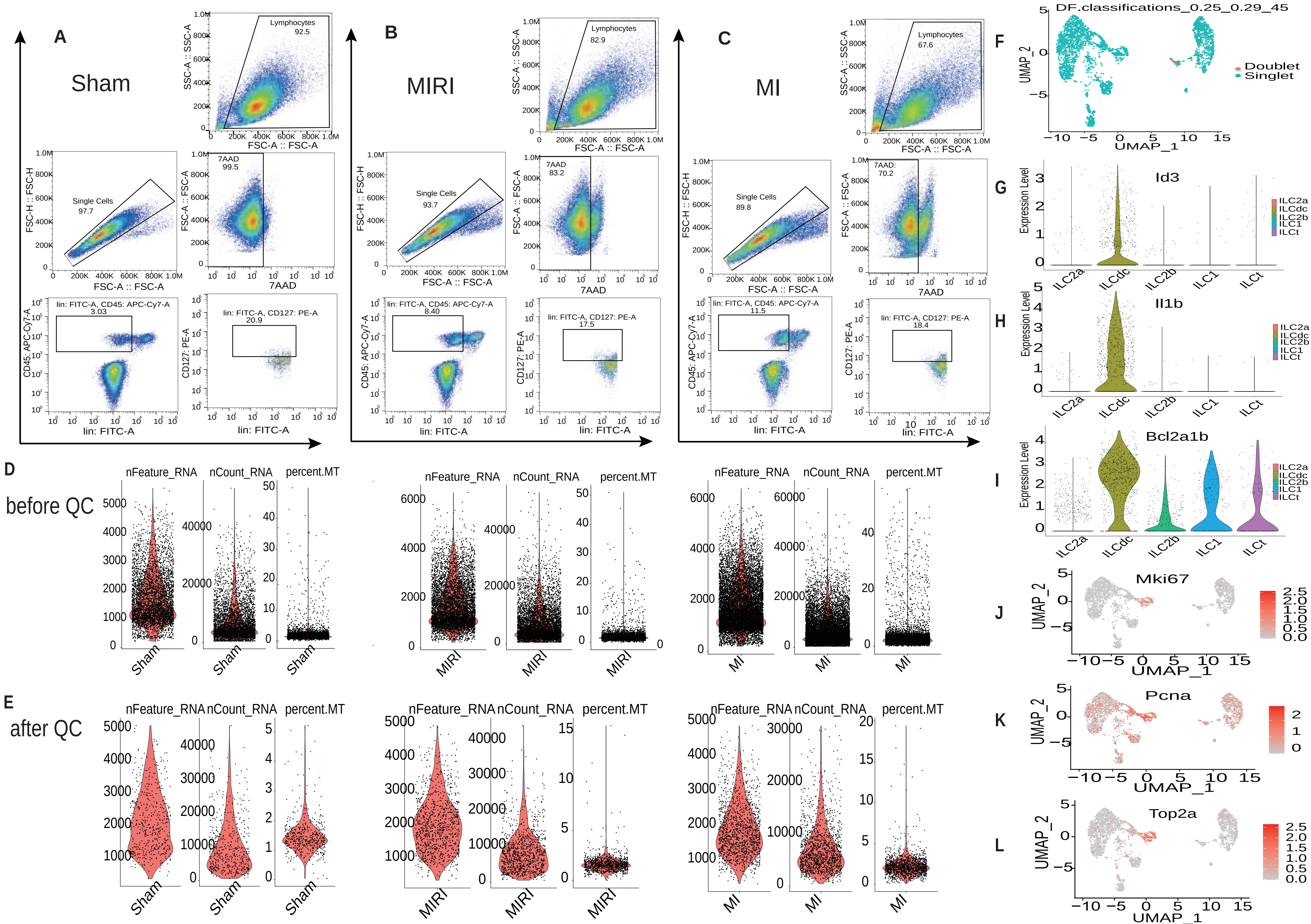
A
Sham
B
MIRI
C
MI
Lymphocytes
92.5
82.9
67.6
SSC-A :: SSC-A
FSC-A :: FSC-A
FSC-H :: FSC-H
Single Cells
97.7
93.7
89.8
7AAD
99.5
83.2
70.2
lin: FITC-A, CD45: APC-Cy7-A
3.03
8.40
11.5
CD45: APC-Cy7-A
lin: FITC-A
lin: FITC-A, CD127: PE-A
20.9
17.5
18.4
CD127: PE-A
D
before QC
E
after QC
nFeature_RNA
nCount_RNA
percent.MT
Sham
MIRI
MI
F
DF.classifications_0.25_0.29_45
Doublet
Singlet
UMAP_1
UMAP_2
G
Id3
H
Il1b
I
Bcl2a1b
Expression Level
ILC2a
ILCdc
ILC2b
ILC1
ILCt
J
Mki67
K
Pcna
L
Top2a

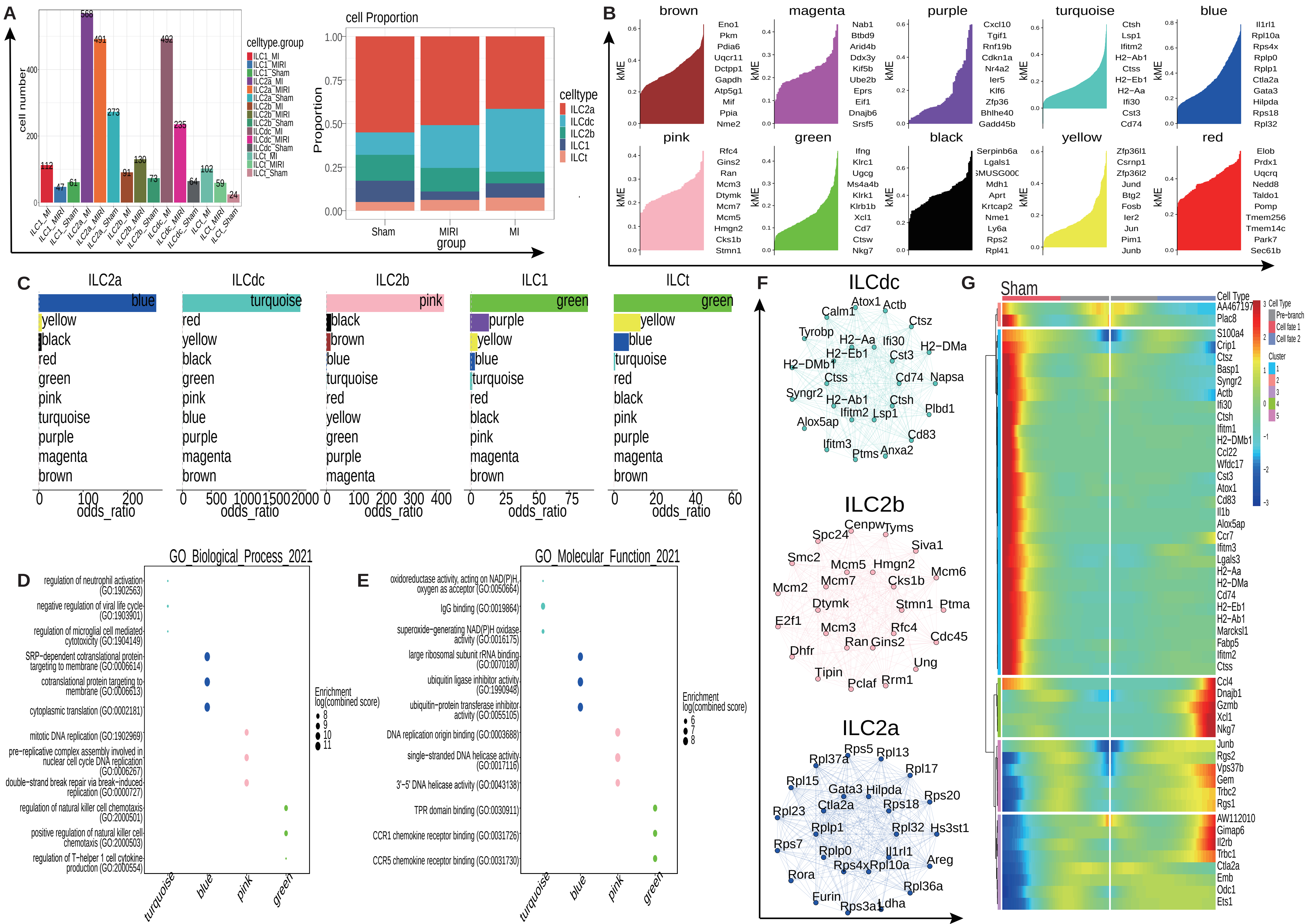
A
cell number
568
491
273
492
235
112
47
61
91
130
73
64
102
59
24
celltype.group
ILC1_MI
ILC1_MIRI
ILC1_Sham
ILC2a_MI
ILC2a_MIRI
ILC2a_Sham
ILC2b_MI
ILC2b_MIRI
ILC2b_Sham
ILCdc_MI
ILCdc_MIRI
ILCdc_Sham
ILCt_MI
ILCt_MIRI
ILCt_Sham
cell Proportion
Proportion
Sham
MIRI
MI
group
celltype
ILC2a
ILCdc
ILC2b
ILC1
ILCt
B
brown
Eno1
Pkm
Pdia6
Uqcr11
Dctpp1
Gapdh
Atp5g1
Mif
Ppia
Nme2
magenta
Nab1
Btbd9
Arid4b
Ddx3y
Kif5b
Ube2b
Eprs
Eif1
Dnajb6
Srsf5
purple
Cxcl10
Tgif1
Rnf19b
Cdkn1a
Nr4a2
Ier5
Klf6
Zfp36
Bhlhe40
Gadd45b
turquoise
Ctsh
Lsp1
Ifitm2
H2-Ab1
Ctss
H2-Eb1
H2-Aa
Ifi30
Cst3
Cd74
blue
Il1rl1
Rpl10a
Rps4x
Rplp0
Rplp1
Ctla2a
Gata3
Hilpda
Rps18
Rpl32
pink
Rfc4
Gins2
Ran
Mcm3
Dtymk
Mcm7
Mcm5
Hmgn2
Cks1b
Stmn1
green
Ifng
Klrc1
Ugcg
Ms4a4b
Klrk1
Klrb1b
Xcl1
Cd7
Ctsw
Nkg7
black
Serpinb6a
Lgals1
MUSG00C
Mdh1
Aprt
Krtcap2
Nme1
Ly6a
Rps2
Rpl41
yellow
Zfp36l1
Csrnp1
Zfp36l2
Jund
Btg2
Fosb
Ier2
Jun
Pim1
Junb
red
Elob
Prdx1
Uqcrq
Nedd8
Taldo1
Pomp
Tmem256
Tmem14c
Park7
Sec61b
kME
C
ILC2a
ILCdc
ILC2b
ILC1
ILCt
odds_ratio
D
GO_Biological_Process_2021
regulation of neutrophil activation (GO:1902563)
negative regulation of viral life cycle (GO:1903901)
regulation of microglial cell mediated cytotoxicity (GO:1904149)
SRP-dependent cotranslational protein targeting to membrane (GO:0006614)
cotranslational protein targeting to membrane (GO:0006613)
cytoplasmic translation (GO:0002181)
mitotic DNA replication (GO:1902969)
pre-replicative complex assembly involved in nuclear cell cycle DNA replication (GO:0006267)
double-strand break repair via break-induced replication (GO:0000727)
regulation of natural killer cell chemotaxis (GO:2000501)
positive regulation of natural killer cell chemotaxis (GO:2000503)
regulation of T-helper 1 cell cytokine production (GO:2000554)
Enrichment log(combined score)
E
GO_Molecular_Function_2021
oxidoreductase activity, acting on NAD(P)H, oxygen as acceptor (GO:0050664)
IgG binding (GO:0019864)
superoxide-generating NAD(P)H oxidase activity (GO:0016175)
large ribosomal subunit rRNA binding (GO:0070180)
ubiquitin ligase inhibitor activity (GO:1990948)
ubiquitin-protein transferase inhibitor activity (GO:0055105)
DNA replication origin binding (GO:0003688)
single-stranded DNA helicase activity (GO:0017116)
3'-5' DNA helicase activity (GO:0043138)
TPR domain binding (GO:0030911)
CCR1 chemokine receptor binding (GO:0031726)
CCR5 chemokine receptor binding (GO:0031730)
turquoise
blue
pink
green
F
ILCdc
ILC2b
ILC2a
G
Sham
Cell Type
Pre-branch
Cell fate 1
Cell fate 2
Cluster

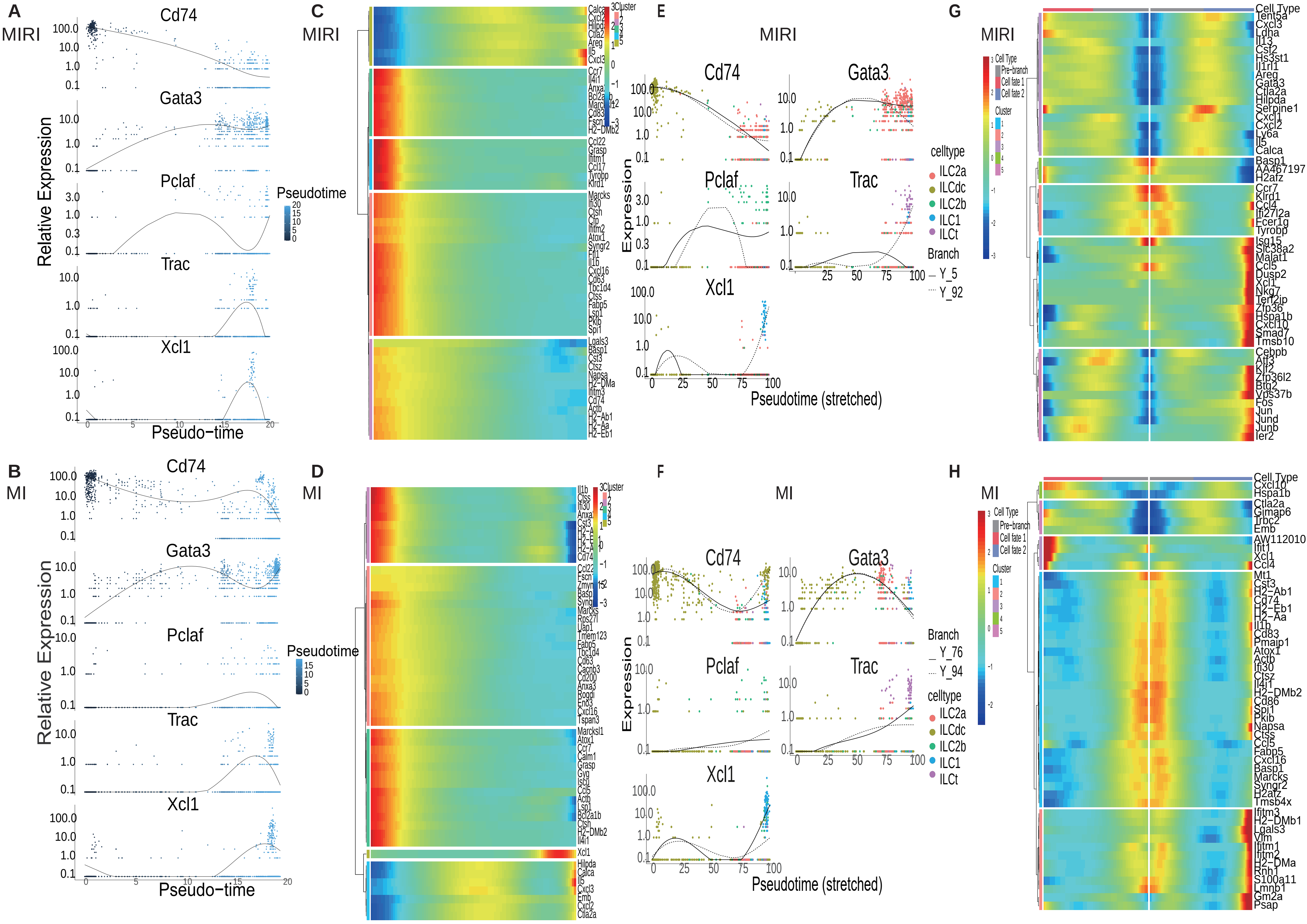
A
MIRI
Cd74
Gata3
Pclaf
Trac
Xcl1
Relative Expression
Pseudo−time
Pseudotime
B
MI
C
MIRI
Cluster
Calca
Cxcl2
Hilpda
Ctla2a
Areg
Il5
Cxcl3
Ccr7
Il4i1
Anxa1
Bcl2a1b
Marcksl1
Cd83
Fscn1
H2−DMb2
Ccl22
Grasp
Ifitm1
Ccl17
Tyrobp
Klrd1
Marcks
Ifi30
Ctsh
Cfp
Ifitm2
Atox1
Syngr2
Ftl1
Il1b
Cxcl16
Cd63
Tbc1d4
Ctss
Fabp5
Lsp1
Pkib
Spi1
Lgals3
Basp1
Cst3
Ctsz
Napsa
H2−DMa
Ifitm3
Cd74
Actb
H2−Ab1
H2−Aa
H2−Eb1
D
MI
Il1b
Ctss
Ifi30
Anxa1
Cst3
Cd74
Ccl22
Fscn1
Basp1
Marcks
Rps27l
Uap1
Tmem123
Fabp5
Tbc1d4
Cd63
Cacnb3
Cd200
Anxa3
Rogdi
Eno3
Cxcl16
Tspan3
Marcksl1
Atox1
Ccr7
Calm1
Grasp
Gyg
Iscu
Ccl5
Actb
Lsp1
Bcl2a1b
Ctsh
H2−DMb2
Il4i1
Xcl1
Hilpda
Calca
Il5
Cxcl3
Emb
Cxcl2
Ctla2a
E
MIRI
Expression
Pseudotime (stretched)
celltype
ILC2a
ILCdc
ILC2b
ILC1
ILCt
Branch
Y_5
Y_92
F
MI
Branch
Y_76
Y_94
G
MIRI
Cell Type
Pre−branch
Cell fate 1
Cell fate 2
Tent5a
Cxcl3
Ldha
Il13
Csf2
Hs3st1
Il1rl1
Areg
Gata3
Ctla2a
Hilpda
Serpine1
Cxcl1
Cxcl2
Ly6a
Il5
Calca
Basp1
AA467197
H2afz
Ccr7
Klrd1
Ccl4
Ifi27l2a
Fcer1g
Tyrobp
Isg15
Slc38a2
Malat1
Ccl5
Dusp2
Xcl1
Nkg7
Terf2ip
Zfp36
Hspa1b
Cxcl10
Smad7
Tmsb10
Cebpb
Atf3
Klf2
Zfp36l2
Btg2
Vps37b
Fos
Jun
Jund
Junb
Ier2
H
MI
Cxcl10
Hspa1b
Ctla2a
Gimap6
Trbc2
Emb
AW112010
Ifit1
Xcl1
Ccl4
Mt1
Cst3
H2−Ab1
Cd74
H2−Eb1
H2−Aa
Il1b
Cd83
Pmaip1
Atox1
Actb
Ifi30
Ctsz
Il4i1
H2−DMb2
Cd86
Spi1
Pkib
Napsa
Ctss
Ccl5
Fabp5
Cxcl16
Basp1
Marcks
Syngr2
H2afz
Tmsb4x
Ifitm3
H2−DMb1
Lgals3
Vim
Ifitm1
Ifitm2
H2−DMa
Rnh1
S100a11
Lmnb1
Gm2a
Psap

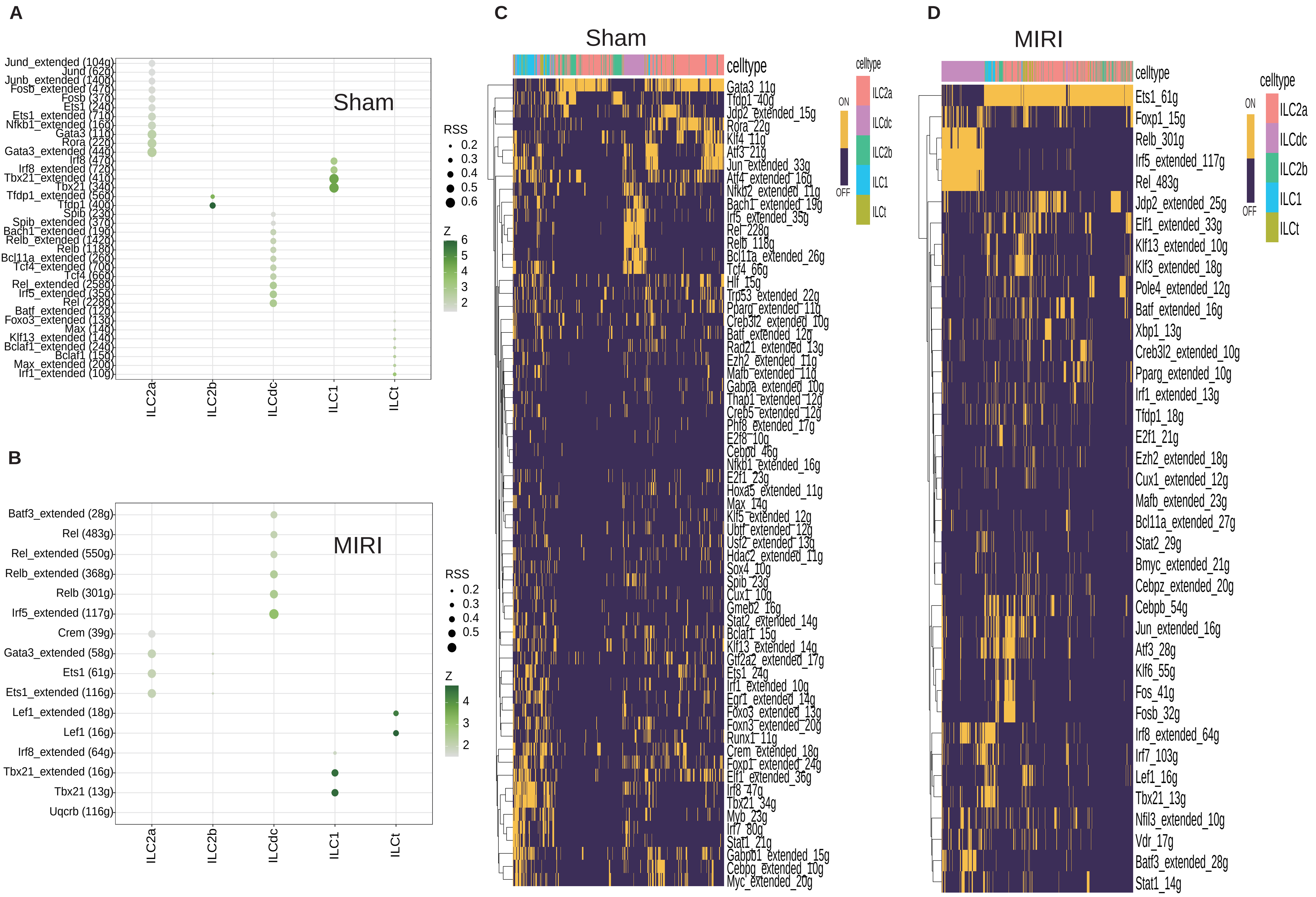
A
Sham
Jund_extended (104g)
Jund (62g)
Junb_extended (140g)
Fosb_extended (47g)
Fosb (37g)
Ets1 (24g)
Ets1_extended (71g)
Nfkb1_extended (16g)
Gata3 (11g)
Rora (22g)
Gata3_extended (44g)
Irf8 (47g)
Irf8_extended (72g)
Tbx21_extended (41g)
Tbx21 (34g)
Tfdp1_extended (56g)
Tfdp1 (40g)
Spib (23g)
Spib_extended (37g)
Bach1_extended (19g)
Relb_extended (142g)
Relb (118g)
Bcl11a_extended (26g)
Tcf4_extended (70g)
Tcf4 (66g)
Rel_extended (258g)
Irf5_extended (35g)
Rel (228g)
Batf_extended (12g)
Foxo3_extended (13g)
Max (14g)
Klf13_extended (14g)
Bclaf1_extended (24g)
Bclaf1 (15g)
Max_extended (20g)
Irf1_extended (10g)
ILC2a
ILC2b
ILCdc
ILC1
ILCt
RSS
0.2
0.3
0.4
0.5
0.6
Z
6
5
4
3
2
B
MIRI
Batf3_extended (28g)
Rel (483g)
Rel_extended (550g)
Relb_extended (368g)
Relb (301g)
Irf5_extended (117g)
Crem (39g)
Gata3_extended (58g)
Ets1 (61g)
Ets1_extended (116g)
Lef1_extended (18g)
Lef1 (16g)
Irf8_extended (64g)
Tbx21_extended (16g)
Tbx21 (13g)
Uqcrb (116g)
ILC2a
ILC2b
ILCdc
ILC1
ILCt
RSS
0.2
0.3
0.4
0.5
Z
4
3
2
C
Sham
celltype
Gata3_11g
Tfdp1_40g
Jdp2_extended_15g
Rora_22g
Klf4_11g
Atf3_21g
Jun_extended_33g
Atf4_extended_16g
Nfkb2_extended_11g
Bach1_extended_19g
Irf5_extended_35g
Rel_228g
Relb_118g
Bcl11a_extended_26g
Tcf4_66g
Hlf_15g
Trp53_extended_22g
Pparg_extended_11g
Creb3l2_extended_10g
Batf_extended_12g
Rad21_extended_13g
Ezh2_extended_11g
Mafb_extended_11g
Gabpa_extended_10g
Thap1_extended_12g
Creb5_extended_12g
Phf8_extended_17g
E2f8_10g
Cebpd_46g
Nfkb1_extended_16g
E2f1_23g
Hoxa5_extended_11g
Max_14g
Klf5_extended_12g
Ubtf_extended_12g
Usf2_extended_13g
Hdac2_extended_11g
Sox4_10g
Spib_23g
Cux1_10g
Gmeb2_16g
Stat2_extended_14g
Bclaf1_15g
Klf13_extended_14g
Gtf2a2_extended_17g
Ets1_24g
Irf1_extended_10g
Egr1_extended_14g
Foxo3_extended_13g
Foxn3_extended_20g
Runx1_11g
Crem_extended_18g
Foxp1_extended_24g
Elf1_extended_36g
Irf8_47g
Tbx21_34g
Myb_23g
Irf7_80g
Stat1_21g
Gabpb1_extended_15g
Cebpg_extended_10g
Myc_extended_20g
celltype
ON
OFF
ILC2a
ILCdc
ILC2b
ILC1
ILCt
D
MIRI
celltype
Ets1_61g
Foxp1_15g
Relb_301g
Irf5_extended_117g
Rel_483g
Jdp2_extended_25g
Elf1_extended_33g
Klf13_extended_10g
Klf3_extended_18g
Pole4_extended_12g
Batf_extended_16g
Xbp1_13g
Creb3l2_extended_10g
Pparg_extended_10g
Irf1_extended_13g
Tfdp1_18g
E2f1_21g
Ezh2_extended_18g
Cux1_extended_12g
Mafb_extended_23g
Bcl11a_extended_27g
Stat2_29g
Bmyc_extended_21g
Cebpz_extended_20g
Cebpb_54g
Jun_extended_16g
Atf3_28g
Klf6_55g
Fos_41g
Fosb_32g
Irf8_extended_64g
Irf7_103g
Lef1_16g
Tbx21_13g
Nfil3_extended_10g
Vdr_17g
Batf3_extended_28g
Stat1_14g
celltype
ON
OFF
ILC2a
ILCdc
ILC2b
ILC1
ILCt

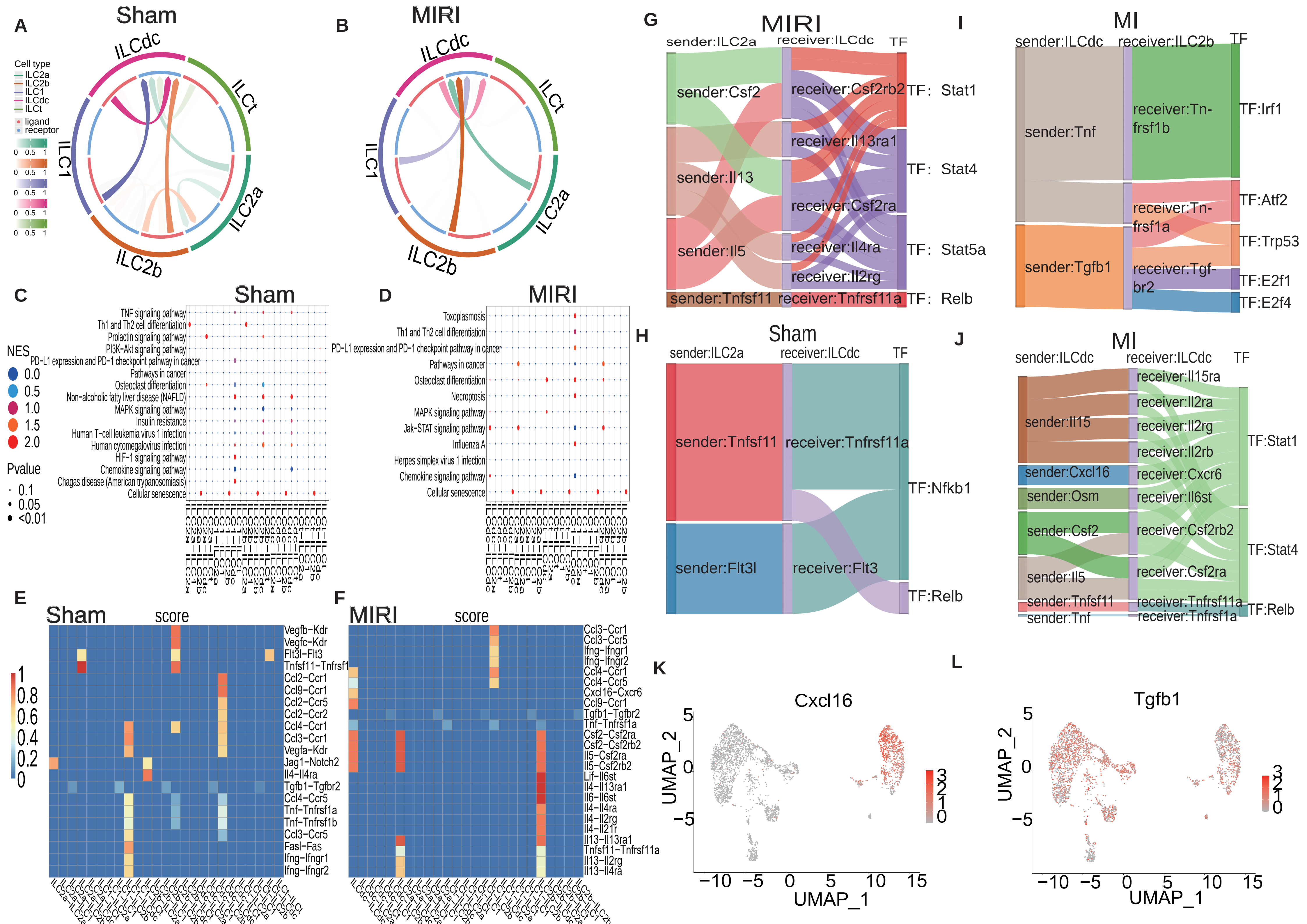
A
Sham
B
MIRI
Cell type
ILC2a
ILC2b
ILC1
ILCdc
ILCt
ligand
receptor
ILCdc
ILCt
ILC1
ILC2a
ILC2b
C
Sham
D
MIRI
NES
0.0
0.5
1.0
1.5
2.0
Pvalue
0.1
0.05
<0.01
TNF signaling pathway
Th1 and Th2 cell differentiation
Prolactin signaling pathway
PI3K−Akt signaling pathway
PD−L1 expression and PD−1 checkpoint pathway in cancer
Pathways in cancer
Osteoclast differentiation
Non−alcoholic fatty liver disease (NAFLD)
MAPK signaling pathway
Insulin resistance
Human T−cell leukemia virus 1 infection
Human cytomegalovirus infection
HIF−1 signaling pathway
Chemokine signaling pathway
Chagas disease (American trypanosomiasis)
Cellular senescence
Toxoplasmosis
Necroptosis
Jak−STAT signaling pathway
Influenza A
Herpes simplex virus 1 infection
E
Sham
score
F
MIRI
Vegfb−Kdr
Vegfc−Kdr
Flt3l−Flt3
Tnfsf11−Tnfrsf11
Ccl2−Ccr1
Ccl9−Ccr1
Ccl2−Ccr5
Ccl2−Ccr2
Ccl4−Ccr1
Ccl3−Ccr1
Vegfa−Kdr
Jag1−Notch2
Il4−Il4ra
Tgfb1−Tgfbr2
Ccl4−Ccr5
Tnf−Tnfrsf1a
Tnf−Tnfrsf1b
Ccl3−Ccr5
Fasl−Fas
Ifng−Ifngr1
Ifng−Ifngr2
Ccl3−Ccr1
Ccl3−Ccr5
Ifng−Ifngr1
Ifng−Ifngr2
Ccl4−Ccr1
Ccl4−Ccr5
Cxcl16−Cxcr6
Ccl9−Ccr1
Tgfb1−Tgfbr2
Tnf−Tnfrsf1a
Csf2−Csf2ra
Csf2−Csf2rb2
Il5−Csf2ra
Il5−Csf2rb2
Lif−Il6st
Il4−Il13ra1
Il6−Il6st
Il4−Il4ra
Il4−Il2rg
Il4−Il21r
Il13−Il13ra1
Tnfsf11−Tnfrsf11a
Il13−Il2rg
Il13−Il4ra
1
0.8
0.6
0.4
0.2
0
G
MIRI
sender:ILC2a
receiver:ILCdc
TF
sender:Csf2
sender:Il13
sender:Il5
sender:Tnfsf11
receiver:Csf2rb2
receiver:Il13ra1
receiver:Csf2ra
receiver:Il4ra
receiver:Il2rg
receiver:Tnfrsf11a
TF: Stat1
TF: Stat4
TF: Stat5a
TF: Relb
H
Sham
sender:ILC2a
receiver:ILCdc
TF
sender:Tnfsf11
sender:Flt3l
receiver:Tnfrsf11a
receiver:Flt3
TF:Nfkb1
TF:Relb
I
MI
sender:ILCdc
receiver:ILC2b
TF
sender:Tnf
sender:Tgfb1
receiver:Tn-frsf1b
receiver:Tn-frsf1a
receiver:Tgf-br2
TF:Irf1
TF:Atf2
TF:Trp53
TF:E2f1
TF:E2f4
J
MI
sender:ILCdc
receiver:ILCdc
TF
sender:Il15
sender:Cxcl16
sender:Osm
sender:Csf2
sender:Il5
sender:Tnfsf11
sender:Tnf
receiver:Il15ra
receiver:Il2ra
receiver:Il2rg
receiver:Il2rb
receiver:Cxcr6
receiver:Il6st
receiver:Csf2rb2
receiver:Csf2ra
receiver:Tnfrsf11a
receiver:Tnfrsf1a
TF:Stat1
TF:Stat4
TF:Relb
K
Cxcl16
UMAP_2
UMAP_1
L
Tgfb1
5
0
−5
−10
10
15
3
2
1
0

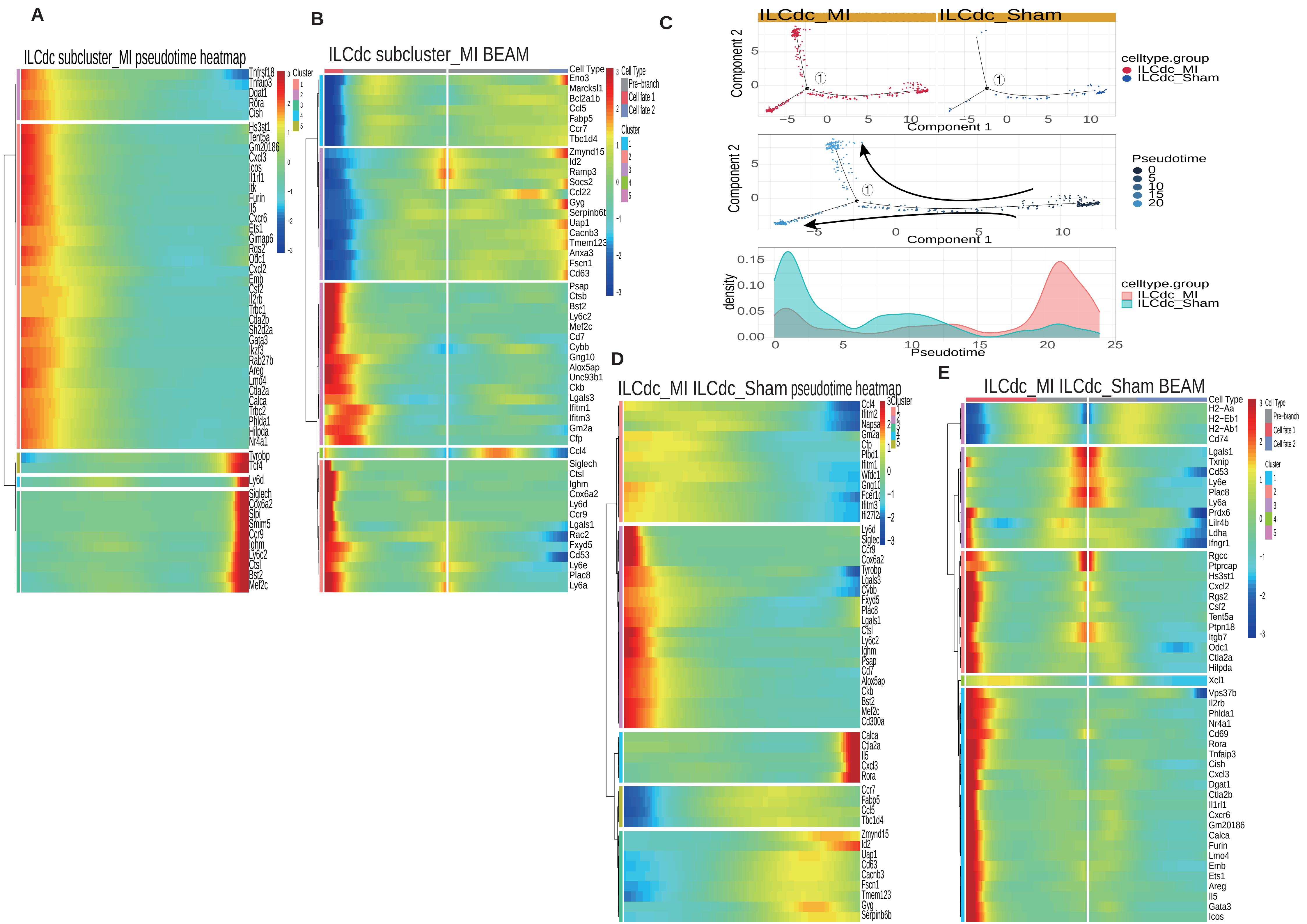
A
ILCdc subcluster_MI pseudotime heatmap
Tnfrsf18
Tnfaip3
Dgat1
Rora
Cish
Hs3st1
Tent5a
Gm20186
Cxcl3
Icos
Il1rl1
Itk
Furin
Il5
Cxcr6
Ets1
Gimap6
Rgs2
Odc1
Cxcl2
Emb
Csf2
Il2rb
Trbc1
Ctla2b
Sh2d2a
Gata3
Ikzf3
Rab27b
Areg
Lmo4
Ctla2a
Calca
Trbc2
Phlda1
Hilpda
Nr4a1
Tyrobp
Tcf4
Ly6d
Siglech
Cox6a2
Slpi
Smim5
Ccr9
Ighm
Ly6c2
Ctsl
Bst2
Mef2c
Cluster
B
ILCdc subcluster_MI BEAM
Cell Type
Eno3
Marcksl1
Bcl2a1b
Ccl5
Fabp5
Ccr7
Tbc1d4
Zmynd15
Id2
Ramp3
Socs2
Ccl22
Gyg
Serpinb6b
Uap1
Cacnb3
Tmem123
Anxa3
Fscn1
Cd63
Psap
Ctsb
Bst2
Ly6c2
Mef2c
Cd7
Cybb
Gng10
Alox5ap
Unc93b1
Ckb
Lgals3
Ifitm1
Ifitm3
Gm2a
Cfp
Ccl4
Siglech
Ctsl
Ighm
Cox6a2
Ly6d
Ccr9
Lgals1
Rac2
Fxyd5
Cd53
Ly6e
Plac8
Ly6a
Cell Type
Pre-branch
Cell fate 1
Cell fate 2
Cluster
C
ILCdc_MI
ILCdc_Sham
Component 2
Component 1
celltype.group
ILCdc_MI
ILCdc_Sham
Pseudotime
0
5
10
15
20
density
Pseudotime
D
ILCdc_MI ILCdc_Sham pseudotime heatmap
Ccl4
Ifitm2
Napsa
Gm2a
Cfp
Plbd1
Ifitm1
Wfdc1
Gng10
Fcer1g
Ifitm3
Ifi27l2a
Ly6d
Siglech
Ccr9
Cox6a2
Tyrobp
Lgals3
Cybb
Fxyd5
Plac8
Lgals1
Ctsl
Ly6c2
Ighm
Psap
Cd7
Alox5ap
Ckb
Bst2
Mef2c
Cd300a
Calca
Ctla2a
Il5
Cxcl3
Rora
Ccr7
Fabp5
Ccl5
Tbc1d4
Zmynd15
Id2
Uap1
Cd63
Cacnb3
Fscn1
Tmem123
Gyg
Serpinb6b
Cluster
E
ILCdc_MI ILCdc_Sham BEAM
Cell Type
H2-Aa
H2-Eb1
H2-Ab1
Cd74
Lgals1
Txnip
Cd53
Ly6e
Plac8
Ly6a
Prdx6
Lilr4b
Ldha
Ifngr1
Rgcc
Ptprcap
Hs3st1
Cxcl2
Rgs2
Csf2
Tent5a
Ptpn18
Itgb7
Odc1
Ctla2a
Hilpda
Xcl1
Vps37b
Il2rb
Phlda1
Nr4a1
Cd69
Rora
Tnfaip3
Cish
Cxcl3
Dgat1
Ctla2b
Il1rl1
Cxcr6
Gm20186
Calca
Furin
Lmo4
Emb
Ets1
Areg
Il5
Gata3
Icos
Cell Type
Pre-branch
Cell fate 1
Cell fate 2
Cluster

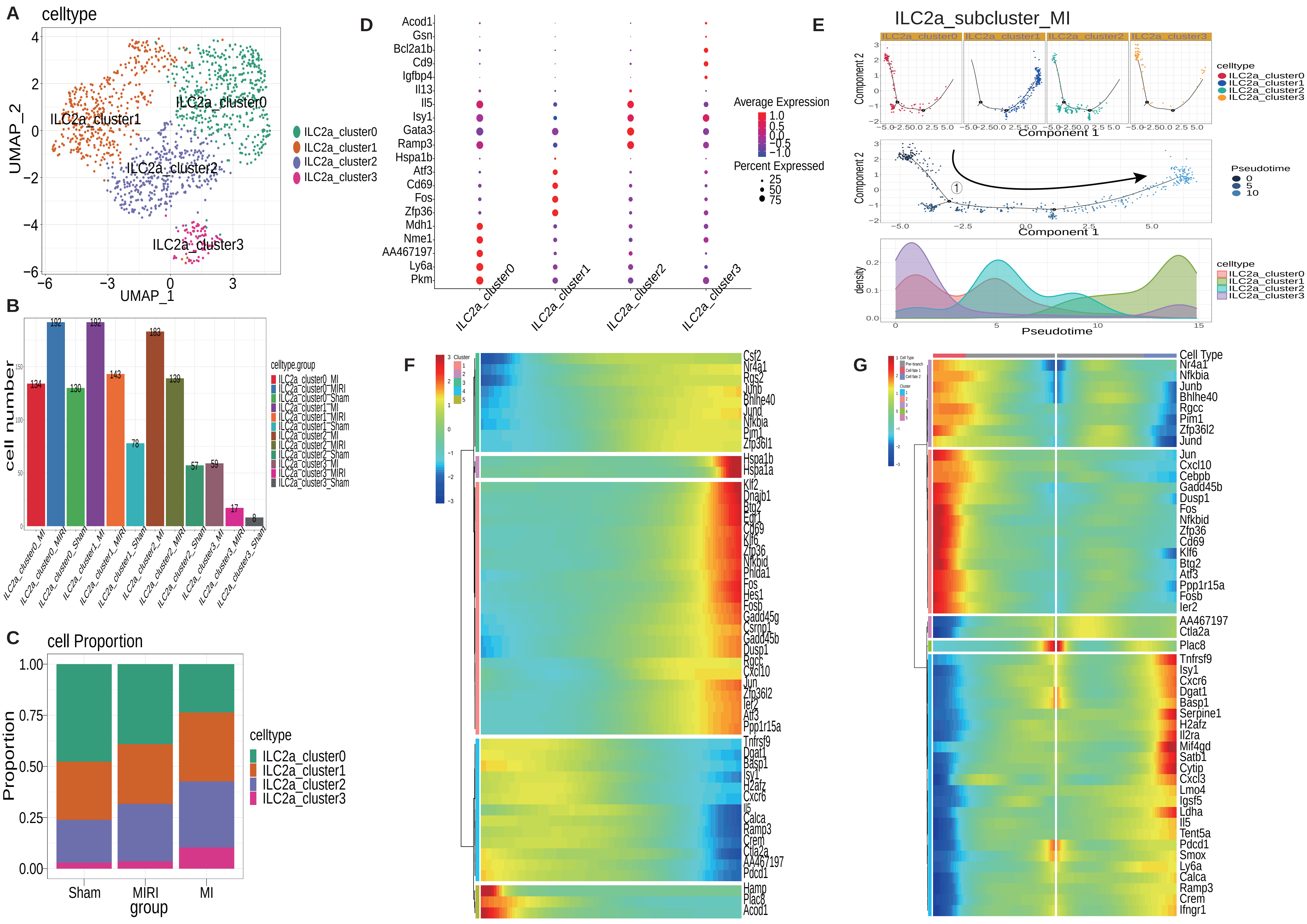
A
celltype
ILC2a_cluster0
ILC2a_cluster1
ILC2a_cluster2
ILC2a_cluster3
UMAP_1
UMAP_2
B
cell number
celltype.group
ILC2a_cluster0_MI
ILC2a_cluster0_MIRI
ILC2a_cluster0_Sham
ILC2a_cluster1_MI
ILC2a_cluster1_MIRI
ILC2a_cluster1_Sham
ILC2a_cluster2_MI
ILC2a_cluster2_MIRI
ILC2a_cluster2_Sham
ILC2a_cluster3_MI
ILC2a_cluster3_MIRI
ILC2a_cluster3_Sham
134 192 130 192 143 78 183 139 57 59 17 8
C
cell Proportion
Proportion
group
Sham
MIRI
MI
celltype
D
Acod1 Gsn Bcl2a1b Cd9 Igfbp4 Il13 Il5 Isy1 Gata3 Ramp3 Hspa1b Atf3 Cd69 Fos Zfp36 Mdh1 Nme1 AA467197 Ly6a Pkm
Average Expression
Percent Expressed
E
ILC2a_subcluster_MI
Component 1
Component 2
Pseudotime
density
F
Cluster
Csf2 Nr4a1 Rgs2 Junb Bhlhe40 Jund Nfkbia Pim1 Zfp36l1 Hspa1b Hspa1a Klf2 Dnajb1 Btg2 Egr1 Cd69 Klf6 Zfp36 Nfkbid Phlda1 Fos Hes1 Fosb Gadd45g Csrnp1 Gadd45b Dusp1 Rgcc Cxcl10 Jun Zfp36l2 Ier2 Atf3 Ppp1r15a Tnfrsf9 Dgat1 Basp1 Isy1 H2afz Cxcr6 Il5 Calca Ramp3 Crem Ctla2a AA467197 Pdcd1 Hamp Plac8 Acod1
G
Cell Type
Pre-branch
Cell fate 1
Cell fate 2
Nr4a1 Nfkbia Junb Bhlhe40 Rgcc Pim1 Zfp36l2 Jund Jun Cxcl10 Cebpb Gadd45b Dusp1 Fos Nfkbid Zfp36 Cd69 Klf6 Btg2 Atf3 Ppp1r15a Fosb Ier2 AA467197 Ctla2a Plac8 Tnfrsf9 Isy1 Cxcr6 Dgat1 Basp1 Serpine1 H2afz Il2ra Mif4gd Satb1 Cytip Cxcl3 Lmo4 Igsf5 Ldha Il5 Tent5a Pdcd1 Smox Ly6a Calca Ramp3 Crem Ifngr1

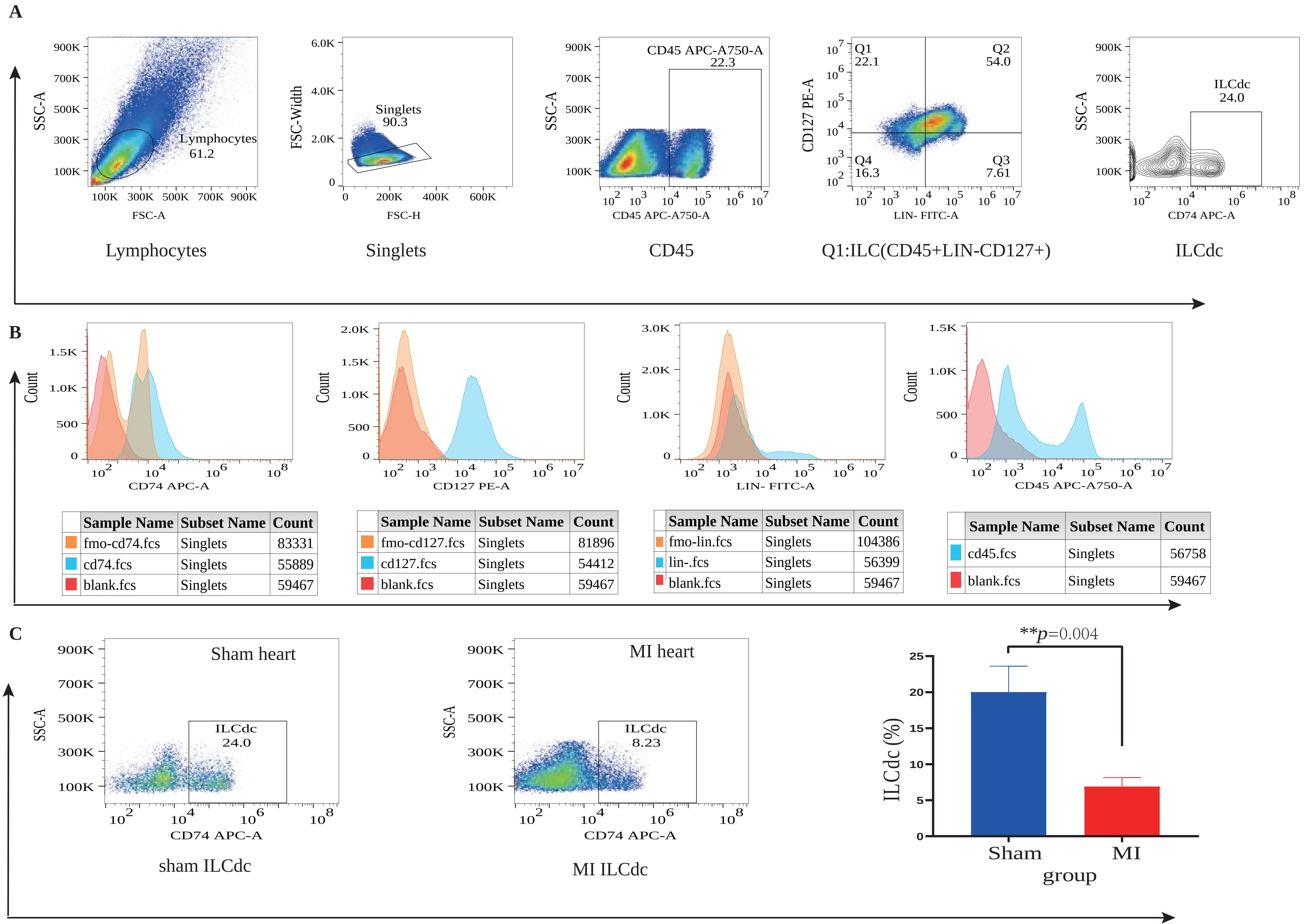

	Sample Name	Subset Name	Count
	fmo-cd74.fcs	Singlets	83331
	cd74.fcs	Singlets	55889
	blank.fcs	Singlets	59467

	Sample Name	Subset Name	Count
	fmo-cd127.fcs	Singlets	81896
	cd127.fcs	Singlets	54412
	blank.fcs	Singlets	59467

	Sample Name	Subset Name	Count
	fmo-lin.fcs	Singlets	104386
	lin-.fcs	Singlets	56399
	blank.fcs	Singlets	59467

	Sample Name	Subset Name	Count
	cd45.fcs	Singlets	56758
	blank.fcs	Singlets	59467

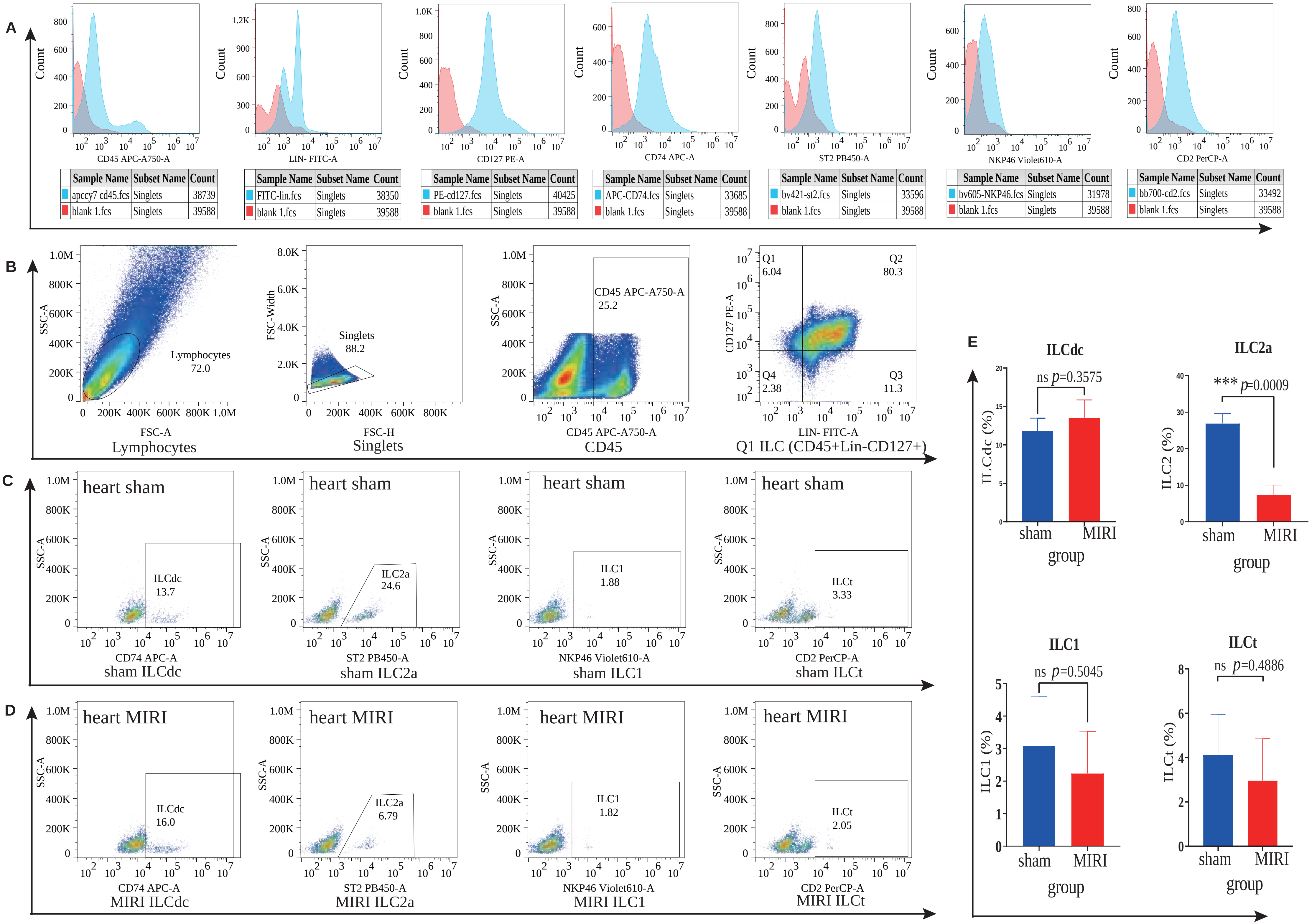

A
CD45 APC-A750-A
LIN- FITC-A
CD127 PE-A
CD74 APC-A
ST2 PB450-A
NKP46 Violet610-A
CD2 PerCP-A
Sample Name | Subset Name | Count
apccy7 cd45.fcs | Singlets | 38739
blank 1.fcs | Singlets | 39588
FITC-lin.fcs | Singlets | 38350
PE-cd127.fcs | Singlets | 40425
APC-CD74.fcs | Singlets | 33685
bv421-st2.fcs | Singlets | 33596
bv605-NKP46.fcs | Singlets | 31978
bb700-cd2.fcs | Singlets | 33492
B
Lymphocytes 72.0
FSC-A
Lymphocytes
Singlets 88.2
FSC-H
Singlets
CD45 APC-A750-A 25.2
CD45
Q1 6.04
Q2 80.3
Q4 2.38
Q3 11.3
LIN- FITC-A
Q1 ILC (CD45+Lin-CD127+)
C
heart sham
ILCdc 13.7
sham ILCdc
ILC2a 24.6
sham ILC2a
ILC1 1.88
sham ILC1
ILCt 3.33
sham ILCt
D
heart MIRI
ILCdc 16.0
MIRI ILCdc
ILC2a 6.79
MIRI ILC2a
ILC1 1.82
MIRI ILC1
ILCt 2.05
MIRI ILCt
E
ILCdc
ns p=0.3575
ILC2a
*** p=0.0009
ILC1
ns p=0.5045
ILCt
ns p=0.4886
sham
MIRI
group

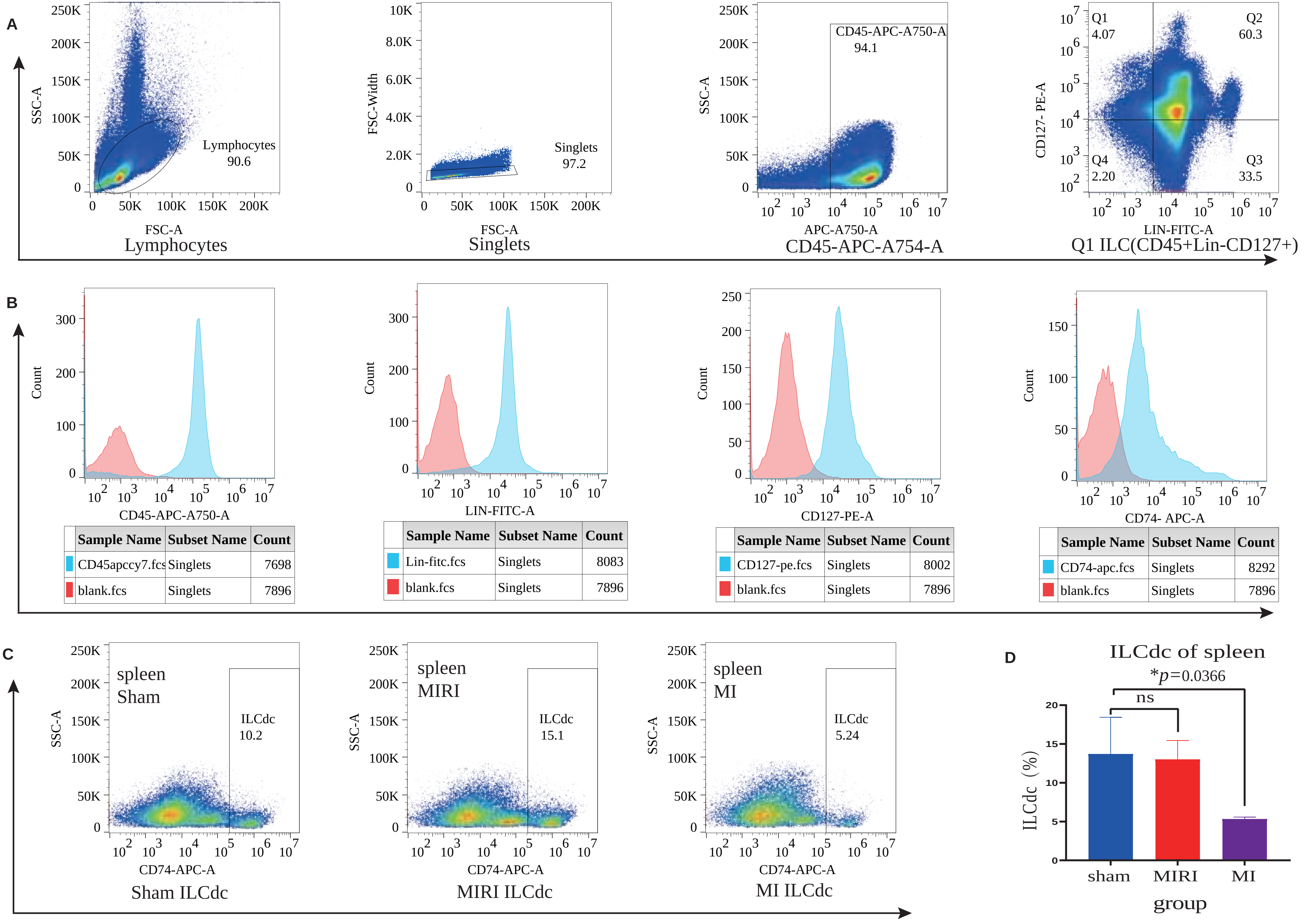
A
SSC-A
FSC-A
Lymphocytes
90.6
Lymphocytes
FSC-Width
Singlets
97.2
Singlets
CD45-APC-A750-A
94.1
APC-A750-A
CD45-APC-A754-A
CD127- PE-A
Q1
4.07
Q2
60.3
Q4
2.20
Q3
33.5
LIN-FITC-A
Q1 ILC(CD45+Lin-CD127+)
B
Count
CD45-APC-A750-A
Sample Name | Subset Name | Count
CD45apccy7.fcs | Singlets | 7698
blank.fcs | Singlets | 7896
LIN-FITC-A
Sample Name | Subset Name | Count
Lin-fitc.fcs | Singlets | 8083
blank.fcs | Singlets | 7896
CD127-PE-A
Sample Name | Subset Name | Count
CD127-pe.fcs | Singlets | 8002
blank.fcs | Singlets | 7896
CD74- APC-A
Sample Name | Subset Name | Count
CD74-apc.fcs | Singlets | 8292
blank.fcs | Singlets | 7896
C
spleen
Sham
ILCdc
10.2
CD74-APC-A
Sham ILCdc
spleen
MIRI
ILCdc
15.1
MIRI ILCdc
spleen
MI
ILCdc
5.24
MI ILCdc
D
ILCdc of spleen
*p=0.0366
ns
ILCdc (%)
sham
MIRI
MI
group

Supplementary figure legends

Fig S1. Flow Sorting, quality control and expression of some specific genes.

A-C. Typical flow cytometry graphs of ILCs sorting ($Lin^{-}CD45^{+}CD127^{+}$) for sham (**A**), MIRI (**B**) and MI group (**C**). **D-E.** Violin plots show the nFeature_RNA, nCount_RNA and percent.MT for different groups before (**D**) and after (**E**) quality control. nFeature_RNA represents the number of genes measured in each cell; nCount_RNA represents the sum of all genes detected in each cell; percent.MT represents the proportion of mitochondrial genes detected and percent. **F.** Remove potential double cells (red dots) with R package DoubletFinder. **G-I.** Violin plots show the expression of ILCreg related genes, such as Id3 (**G**), Il1b **(H**) and Bcl2a1b (**I**). **J-L.** Feature plots show the expression of proliferation-related genes, such as Mki67 (**J**), Pcna (**K**) and Top2a (**L**).

Fig S2. Proportion changes, hdWGCNA analysis of hub genes, pathways and regulatory networks, BEAM analysis.

A. Bar plot visualizing the number of ILCs clusters in different disease groups, and stack plot visualizing the proportion of ILCs clusters in different disease groups. **B.** Top 10 genes in each module were identified by hdWGCNA analysis according to kME value. **C.** The modules are reassigned into 5 new clusters by “OverlapModulesDEGs” function and then annotate the 5 clusters by DEGs in ILCs. **D-E.** GO Biological process was shown in (**D**) and molecular function was shown in (**E**) to exhibit signal enrichments in special

modules. **F.** Network plot shows the hub genes of ILCdc (turquoise module), ILC2a (blue module) and ILC2b (pink module). **G.** Heatmap of the top 50 differential genes(P<0.01) discovered in branch point 1 of sham group by the BEAM function.

Fig S3. Pseudotime analysis of ILCs clusters differentiation in MIRI and MI group.

A-B. Change trend of Cd74, Gata3, Pclaf, Trac and Xcl1 on pseudotime trajectory in the MIRI (**A**) and MI group (**B**). **C-D.** Displaying top 50 differential genes of pseudotime variation (P<0.01) by clustering heatmap in the MIRI group (**C**) and MI group (**D**). **E-F.** Pseudotime kinetics of Cd74, Gata3, Pclaf, Trac and Xcl1 from the root to two branch points in the MIRI group (**E**) and MI group (**F**). **G-H.** Heatmap of the top 50 differential genes (P<0.01) discovered in branch point 1 of MIRI group (**G**) and MI group (**H**) by the "BEAM" function with "monocle2".

Fig S4. Analysis of ILCs clusters TF in sham and MIRI by SCENIC.

A-B. Use RSS to identify ILCs clusters specific regulons in the sham group (**A**) and in the MIRI group (**B**). The color depth represents the z-score value, and the size of the point represents the RSS score. **C-D.** Heatmaps show the binary converted regulators activated in the sham group (**C**) and in the MIRI group (**D**).

Fig S5. Intercellular communication among ILCs clusters in sham and MIRI groups.

A-B. Circos plots show the interactions of ligand-receptors across and within ILCs clusters in sham (**A**) and MIRI group (**B**). **C-D.** Bubble plots show the enrichment pathways of paired ligand-receptor data sets between signal sender and receiver in sham (**C**) and MIRI group (**D**). **E-F.** Heatmaps show the paired ligand-receptor involved between the signal sender and receiver cells in sham (**E**) and MIRI group (**F**). **G-H.** Sankey plots display the cell communications between ILC2a and ILCdc and downstream TFs in sham group (**G**) and MIRI group (**H**). **I.** Sankey plot displays the cell communications between ILCdc and ILC2b and downstream TFs in MI group**. J.** Sankey plot displays the cell communications between ILCdc and ILCdc and downstream TFs in MI group. **K-L.** Feature plots show the expression of Cxcl16 and Tgfb1.

Fig S6. Pseudotime analysis of ILCdc differentiation.

A. Heatmap displays several gene cohorts that changed during the pseudotime transition of the ILCdc sub-clusters in MI group. **B.** Heatmap of Top 50 significantly changed genes (P<0.01) discovered by the BEAM function with monocle2 at branch point 1 of the ILCdc subclusters in MI group. **C.** Pseudotime analysis of ILCdc development and distribution between MI group and Sham group. **D.** Heatmap displays several gene cohorts that changed during the pseudotime transition in the ILCdc between MI group and Sham group. **E.** Heatmap of Top 50 significantly changed genes (P<0.01) discovered by the BEAM function with monocle2 at branch point 1 of the ILCdc between MI group and Sham group.

Fig S7. The heterogeneities and transcriptional features of ILC2a.

A. Visualization of ILC2a Sub-clusters Infiltrated in ischemic Myocardium of Mice by UMAP. **B.** Bar plot shows the cell number of ILC2a sub-clusters in different disease groups. **C.** Stack plot visualizes the proportion of ILC2a Sub-clusters in different disease groups. **D.** Dot plot displays the marker genes of ILC2a Sub-clusters. **E.** Distribution statuses of ILC2a subclusters on pseudotime trajectory in the MI group. **F.** Heatmap displays several gene cohorts that changed during the pseudotime transition of the ILC2a sub-clusters in MI group. **G.** Heatmap of Top 50 significantly changed genes ($P<0.01$) discovered by the BEAM function with monocle2 at branch point 1 of the ILC2a subclusters in MI group.

Fig S8. Identification of ILCdc in cardiac mononuclear cells by flow cytometry

A. Flow cytometric gating strategy of ILCdc ($Lin^{-}CD45^{+}CD127^{+}CD74^{+}$) in cardiac mononuclear cells, Q1 represents ILC ($Lin^{-}CD45^{+}CD127^{+}$). **B.** The blank control fluorescence channel and the single antibody labeled fluorescence channel are shown by the histogram, red color represents the blank control fluorescence channel, blue color represents the CD74-APC, CD127-PE, Lin-FITC, CD45-APC-A750 fluorescence channel, respectively. The orange color represents the FMO control for CD74-APC, CD127-PE, and Lin-FITC, respectively. **C.** ILCdc($Lin^{-}CD45^{+}CD127^{+}CD74^{+}$) was identified in cardiac mononuclear cells by flow cytometry, and the proportion of ILCdc in

the heart was shown in sham and MI groups, respectively. **D.** Comparison of ILCdc differences between sham and MI groups by two-tailed unpaired T test, n=3, 3-5 mice per sample, *represents a significant difference (p<0.05), * *represents a significant difference (p<0.01), ns represents no significant difference.

Fig S9. ILC subsets in cardiac mononuclear cells in MIRI and sham group were identified by flow cytometry

A. The blank control fluorescence channel and single antibody labeled fluorescence channel are shown by histogram, red color represents the blank control fluorescence channel, The blue color represents the fluorescence channels of CD45-APC-A750, Lin-FITC, CD127-PE, CD74-APC, ST2-PB450, NKP46-Violet610, and CD2-Percp, respectively. The X-axis represents the value of the fluorescence channel, and the Y-axis represents the number of cells. **B.** Flow cytometric gating strategy of ILC(Lin^-CD45^+ $CD127^+$) in cardiac mononuclear cells, Q1 represents ILC($Lin^-CD45^+CD127^+$). **C-D.** Identification of ILCdc($Lin^-CD45^+CD127^+CD74^+$), LC2a($Lin^-CD45^+CD127^+ST2^+$), LC1 ($Lin^-CD45^+CD127^+NKP46^+$), ILCt($Lin^-CD45^+CD127^+CD2^+$) in cardiac mononuclear cells by flow cytometry, Represents the proportions of ILCdc, ILC2a, ILC1, and ILCt in sham and MIRI groups, respectively. **E.** Comparison of ILCdc, ILC2a, ILC1, and ILCt between sham and MIRI groups by two-tailed unpaired T test, respectively, n=3, 3-5 mice per sample. * represents a significant difference (p<0.05), ** represents a significant difference (p<0.01), *** represents a significant difference (p<0.001), ****represents a

significant difference (p<0.0001), ns represents no significant difference.

Fig S10. ILCdc in splenic mononuclear cells was identified by flow cytometry

A. Flow cytometric gating strategy of ILCs ($Lin^-CD45^+CD127^+$) on splenic mononuclear cells, Q1 represents ILCs ($Lin^-CD45^+CD127^+$). **B.** The blank control fluorescence channel and single antibody labeled fluorescence channel are shown by histogram, red color represents the blank control fluorescence channel, blue color represents the CD45-APC-A750, Lin-FITC, CD127-PE, CD74-APC fluorescence channel respectively, x axis represents the value of fluorescence channel, y axis represents the number of cells. **C.** ILCdc($Lin^-CD45^+CD127^+CD74^+$) in splenic mononuclear cells was identified by flow cytometry, showing the proportion of ILCdc in sham, MIRI, and MI groups, respectively, and the gating strategy represents $CD74^+$ cells from Q1 ILC($Lin^-CD45^+CD127^+$). **D.** Comparison of ILCdc differences among sham, MIRI, and MI groups by one-way ANOVA, n=3, 3-5 mice per sample, * represents a significant difference ($p<0.05$), ns represents no significant difference.
