# Supplementary material for: Single-cell landscape dissecting the transcription and heterogeneity of innate lymphoid cells in ischemic heart
Source: Front Immunol. 2023 May 9;14:1129007. doi: 10.3389/fimmu.2023.1129007 (PMC10203554; doi:10.3389/fimmu.2023.1129007)
Supplement: Supplementary file 5 [file DataSheet_5.doc]

| **REAGENT or RESOURCE** | **SOURCE** | **IDENTIFIER** |
| --- | --- | --- |
| **1.Antibodies** |  |  |
| Mouse Hematopoietic Lineage Antibody Cocktail, FITC, eBioscience™ | Invitrogen | Cat#22-7770-72 |
| APC/Cyanine7 anti-mouse CD45 Antibody | BioLegend | Cat#103116 |
| PE anti-mouse CD127 (IL-7Rα) Antibody | BioLegend | Cat#121112 |
| eBioscience™ 7-AAD Viability Staining S Solution | Invitrogen | Cat#00-6993 |
| Alexa Fluor® 647 anti-mouse CD74 (CLIP) Antibody | BioLegend | Cat#151004 |
| BV421 Rat Anti-Mouse IL-33R (ST2) | BD Biosciences | Cat#566310 |
| BB700 Rat Anti-Mouse CD2 | BD Biosciences | Cat#742090 |
| BV605 Rat Anti-Mouse CD335 (NKp46) (29A1.4) | BD Biosciences | Cat#564069 |
| **2.Chemicals** |  |  |
| Collagenase B | Sigma | Cat#11088815001 |
| Percoll | Solarbio | Cat#P837 |
| RPMI 1640 | Hyclone | Cat#SH30096.FS |
| Single Cell Sequence Specific Amplification Kit | Vazyme Biotech Co., Ltd | Cat#P621 |
| AceQ® qPCR SYBR Green Master Mix | Vazyme Biotech Co., Ltd | Cat#Q111 |
| **3. Primers** | | |
| GAPDH-F | Tsingke Biotechnology Co., Ltd. | AGGTCGGTGTGAACGGATTTG |
| GAPDH-R | Tsingke Biotechnology Co., Ltd. | TGTAGACCATGTAGTTGAGGTCA |
| IL5-F | Tsingke Biotechnology Co., Ltd. | CTCTGTTGACAAGCAATGAGACG |
| IL5-R | Tsingke Biotechnology Co., Ltd. | TCTTCAGTATGTCTAGCCCCTG |
| IL13-F | Tsingke Biotechnology Co., Ltd. | CCTGGCTCTTGCTTGCCTT |
| IL13-R | Tsingke Biotechnology Co., Ltd. | GGTCTTGTGTGATGTTGCTCA |
| Relb-F | Tsingke Biotechnology Co., Ltd. | CCGTACCTGGTCATCACAGAG |
| Relb-R | Tsingke Biotechnology Co., Ltd. | CAGTCTCGAAGCTCGATGGC |
| Ciita-F | Tsingke Biotechnology Co., Ltd. | GGAGGAGATCGAACTCAGCTC |
| Ciita-R | Tsingke Biotechnology Co., Ltd. | GTTCCGCAATGTTGGCATAGG |
| IL21r-F | Tsingke Biotechnology Co., Ltd. | GGCTGCCTTACTCCTGCTG |
| IL21r-R | Tsingke Biotechnology Co., Ltd. | TCATCTTGCCAGGTGAGACTG |
| Ifngr2-F | Tsingke Biotechnology Co., Ltd. | TCCTCGCCAGACTCGTTTTC |
| Ifngr2-R | Tsingke Biotechnology Co., Ltd. | GTCTTGGGTCATTGCTGGAAG |
| Tnf-F | Tsingke Biotechnology Co., Ltd. | CCCTCACACTCAGATCATCTTCT |
| Tnf-R | Tsingke Biotechnology Co., Ltd. | GCTACGACGTGGGCTACAG |
| IL6ra-F | Tsingke Biotechnology Co., Ltd. | CCTGAGACTCAAGCAGAAATGG |
| IL6ra-R | Tsingke Biotechnology Co., Ltd. | AGAAGGAAGGTCGGCTTCAGT |
| IL4ra-F | Tsingke Biotechnology Co., Ltd. | TCTGCATCCCGTTGTTTTGC |
| IL4ra-R | Tsingke Biotechnology Co., Ltd. | GCACCTGTGCATCCTGAATG |
| IL4-F | Tsingke Biotechnology Co., Ltd. | GGTCTCAACCCCCAGCTAGT |
| IL4-R | Tsingke Biotechnology Co., Ltd. | GCCGATGATCTCTCTCAAGTGAT |
| Ifng-F | Tsingke Biotechnology Co., Ltd. | ATGAACGCTACACACTGCATC |
| Ifng-R | Tsingke Biotechnology Co., Ltd. | CCATCCTTTTGCCAGTTCCTC |
| Tnfsf11-F | Tsingke Biotechnology Co., Ltd. | CAGCATCGCTCTGTTCCTGTA |
| Tnfsf11-R | Tsingke Biotechnology Co., Ltd. | CTGCGTTTTCATGGAGTCTCA |
| **4. equipment** | | |
| Cell Sorter SH-800 LE-B3001 | Sony Biotechnology Inc. |  |
| Cell Sorter Beckman Moflo-XDP | Beckman Coulter, Inc. |  |
| Beckman Cytoflex S | Beckman Coulter Biotechnology (Suzhou) Co.,Ltd |  |
| **5.Software and algorithms** | | |
| GraphPad Prism (v9.0.0) | GraphPad | https://www.graphpad.com/ |
| FlowJo (v10.5.3) | BD Biosciences | https://www.flowjo.com/ |
| RStudio (v4.1.1) | RStudio, Inc. | https://www.rstudio.com/ |
| Seurat (v4.1.1) | (Stuart et al., 2019) | <https://github.com/satijalab/seurat> |
| SCENIC(v1.3.1) | (Aibar et al., 2017) | https://github.com/aertslab/SCENIC |
| cellcall(v0.0.0.9000) | ([ShellyCoder](https://github.com/ShellyCoder),2020) | https://github.com/ShellyCoder/cellcall |
| Monocle(v2.22.0) | (Cole Trapnell,2014) | http://cole-trapnell-lab.github.io/monocle-release/docs/ |
| hdWGCNA(0.1.1.9010) | ([Sam Morabito](https://smorabit.github.io/),2021) | https://smorabit.github.io/hdWGCNA/index.html |
| SingleR(v1.8.1) | (Aran et al., 2019) | https://github.com/dviraran/SingleR |
| ggplot2(v3.3.6) | Tidyverse | https://github.com/tidyverse/ggplot2/ |
| EnhancedVolcano(v1.12.0) | (Blighe, K, S Rana, and M Lewis. 2018) | [https://github.com/kevinblighe/EnhancedVolcano](https://github.com/kevinblighe/EnhancedVolcano.) |
| GSVA(v1.42.0) | (Castelo R,2013) | https://github.com/rcastelo/GSVA |
| clusterProfiler(4.2.2) | (G Yu,2012) | https://yulab-smu.github.io/clusterProfiler-book |
| pheatmap | Raivo Kolde | https://github.com/raivokolde/pheatmap |
| Software and algorithms | Software |  |
| GraphPad Prism (v9.0.0) | GraphPad | https://www.graphpad.com/ |
